# Excited State Transient Phenomena in Two Different Phases of the Photoactive MOF MIP‐177(Ti)

**DOI:** 10.1002/smll.202407273

**Published:** 2025-02-19

**Authors:** Aneek Kuila, Valentin Diez‐Cabanes, Arianna Melillo, Jonas Gosch, Amarajothi Dhakshinamoorthy, Shilin Yao, Georges Mouchaham, Christian Serre, Hermenegildo García, Sergio Navalon, James R. Durrant, Guillaume Maurin, Yaron Paz

**Affiliations:** ^1^ Department of Chemical Engineering Technion Haifa 3200003 Israel; ^2^ ICGM Université de Montpellier CNRS ENSCM Montpellier 34293 France; ^3^ Institut des Matériaux Poreux de Paris ESPCI Paris École Normale Supérieure CNRS PSL University Paris 75005 France; ^4^ Instituto de Tecnología Química (UPV‐CSIC) Universitat Politècnica de València Avenida de los Naranjos s/n Valencia 46022 Spain; ^5^ Departamento de Química Universitat Politècnica de València Camino de Vera s/n Valencia 46022 Spain; ^6^ Department of Chemistry and Centre for Processable Electronics Imperial College London London W12 0BZ UK

**Keywords:** charge life‐time, MIP‐177(Ti), MOF, photocatalytic hydrogen evolution reaction, time‐dependent density functional perturbation theory simulation, transient IR spectroscopy, UV–Vis diffuse‐reflectance transient absorption spectroscopy

## Abstract

The metal organic framework (MOF) MIP‐177(Ti) is under the spotlight for its robust photo‐response and stability. This MOF can be synthesized in forms: MIP‐177(Ti)‐LT (LT: low temperature) and MIP‐177(Ti)‐HT (HT: high temperature). The MIP‐177(Ti)‐LT version comprises of Ti_12_O_15_ units interconnected by 3,3′,5,5′‐tetracarboxydiphenylmethane (mdip) ligands and interconnecting formate groups. Upon high temperature treatment, MIP‐177(Ti)‐LT loses its formate groups, thus rearranging into a continuous 1‐D chain of Ti_6_O_9_ units leading to the MIP‐177(Ti)‐HT. Based on this 1‐D connected structure, one should expect a higher catalytic activity of MIP‐177(Ti)‐HT. Nevertheless, the hydrogen evolution reaction photoactivity assessment clearly indicates the opposite. Combining transient IR measurements (TRIR), TAS and DFT/TD‐DFPT calculations unveils the reasons for this situation. The TRIR measurements evidence that the photoinduced electrons are located in the inorganic part, while the holes are in the mdip ligand. The longer lifetime of MIP‐177(Ti)‐LT is mapped onto a slower decay of the Ti–O related peaks. A reversible change in the coordination of the carboxylate groups from a bidentate to a monodentate coordination is observed only in MIP‐177(Ti)‐LT. Complementary DFT and TD‐DFPT simulations demonstrate a higher electron delocalization on the inorganic part for MIP‐177(Ti)‐LT (hence, enhanced mobility and slower recombination), thus explaining its superior photocatalytic activity.

## Graphical abstract

MIP‐177(Ti)‐high temperature (HT) and MIP‐177(Ti)‐low temperature (LT) are stable, photoresponsive metal organic frameworks (MOFs). Despite its structural advantages, MIP‐177(Ti)‐HT has lower hydrogen evolution reaction photoactivity than MIP‐177(Ti)‐LT. Advanced time dependent spectroscopies and theoretical models reveal that MIP‐177(Ti)‐LT demonstrates a long lasting, reversible coordination change of carboxylate coordination and enhanced electron delocalization, which contribute to its superior photocatalytic performance.

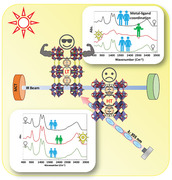

## 1 Introduction

The paradigm shifts in the research on the photoactive porous materials to produce solar fuels contributes to fulfilling the international commitment to achieve net zero carbon emission. Unfortunately, molecular catalysis involving current porous materials might suffer from low surface area, fast inactivation and photoinduced self‐degradation, which limit large‐scale application. The use of metal organic frameworks (MOFs) consisting of ordered and porous hybrid organic‐inorganic scaffold with well‐defined active sites may tackle these drawbacks by providing large surface area for adsorption/diffusion of reactants/products together with increased lifetime of photogenerated charge carriers due to their spatial separation.^[^ ^1^ ^]^ The properties of these hybrid crystalline materials can be fine‐engineered almost at will by introducing a broad range of metal ions and organic secondary building units or by performing post‐synthesis modifications to address specific applications. Owing to the relatively high strength of the metal–ligand bond in MOFs, the open metal sites are usually considered unaffected during photoexcitation.^[^ ^2^ ^]^ This suggests that the coordination bonds of MOFs may maintain their configuration at the ground state upon excitation. In contrast, several reports propose that the metal–ligand coordination bond may change following excitation and that the dynamic nature of the coordination bond may explain several observed properties of MOF glasses.^[^ ^3^ ^]^

Time resolved infrared absorption spectroscopy (TRIR) supported by density functional theory (DFT) and time‐dependent density functional perturbation theory (TD‐DFPT) calculations may help in tracking structural changes in the excited state. In general, TRIR provides valuable insights about the interactions between trapped charges and adjacent nuclei in photoactive materials by following subtle spectral changes such as shifts, appearance of new transitions and variations in intensities of existing peaks due to polarizability alterations.^[^ ^4^ ^]^ As an example, relevant to MOFs, the dynamic changes in the coordination between metals and carboxylates may change the bond order, leading to transient changes in specific vibrational frequencies.^[^ ^5^ ^]^

**Figure 1:**
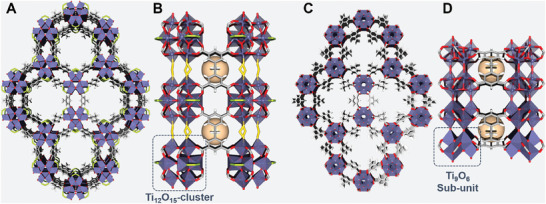
Crystal structures of A,B) MIP‐177(Ti)‐LT and C,D) MIP‐177(Ti)‐HT: Overall views (A, C) and close up cuts along the channels, revealing the linkage between the inorganic and organic building units. Color code: Ti‐ purple polyhedral; C—gray, O—red; H—white; peripherical formats—green; bridging formats—yellow. Orange spheres represent the small pockets between two MDIP linkers.

This study focuses on a recently developed Ti‐based MOF denoted as MIP‐177(Ti)‐LT (MIP stands for Materials from Institute of Porous Materials of Paris (LT: low temperature) in comparison with its high temperature form, denoted MIP‐177(Ti)‐HT (HT: High Temperature) obtained after thermal treatment.^[^ ^6^ ^]^ MIP‐177(Ti) is under the spotlight for its superior photo‐response and stability. The d° configuration of the metal sites, arising from the presence of Ti^4+^ in the inorganic node, similarly to other Ti‐MOFs,^[^ ^7^ ^]^ does not exhibit the d–d electronic excitation/relaxation, considered to be the most prominent deactivation pathway of open shell transition metals, that hampers their photoactivity.^[^ ^8^ ^]^ More specifically, the microporous structure of this MIP‐177(Ti)‐LT originates from the coordination of Ti_12_O_15_ units to secondary building units of 3,3′,5,5′‐tetracarboxydiphenylmethane (mdip). Its honeycomb‐like crystal structure has the formula of Ti_12_O_15_(mdip)_3_(formate)_6_ where the Ti (IV) ions are interconnected by µ_3_‐oxo bridges which are further connected with carboxylate groups of the spacers and half of the formates (**Figure** **1**A). MIP‐177(Ti)‐LT experiences a topotactical transformation under high temperature and converted to its MIP‐177(Ti)‐HT form due to the decomposition of formats and their departure, resulting in rearrangement of the Ti─O clusters into a continuous 1D chain of Ti_6_O_9_ units, while the honeycomb arrangement remains very similar (Figure 1B).^[^ ^6^, ^9^ ^]^ This unusual reorganization was associated, as determined by time resolved microwave conductivity (TRMC) analysis, with a drastic increase in the charge separation life time for the MIP‐177(Ti)‐HT compared to the oxocluster based form MIP‐177(Ti)‐LT, further extended to the ms time scale once loaded with a conductive polymer.^[^ ^6^ ^]^

Among the two MIP‐177(Ti)s, the MIP‐177(Ti)‐LT version has gained considerable interest owing to its exceptional photo responsive characteristic and high chemical stability under extremely high acidic environments.^[^ ^10^ ^]^ Indeed, it was shown to tolerate both aqua regia and concentrated phosphoric acid,^[^ ^11^ ^]^ while its exposure to an aqueous solution of formic acid (FA) under visible light illumination led to hydrogen production (from the decarboxylation of FA), significantly higher than other Ti‐containing MOFs like MIL‐125(Ti)‐NH_2_ and the reference TiO_2_ (P25).^[^ ^12^ ^]^ The highly stable nature of MIP‐177(Ti)s has also made them suitable candidates for nitric oxide delivery systems in simulated body fluids or as materials for proton exchange membranes for fuel cell once sulfonated with sulfuric acid.^[^ ^13^ ^]^

Herein, we report a combination of advanced spectroscopies (time‐resolved IR and diffuse‐reflectance UV–Vis transient absorption) in tandem with TD‐DFPT simulations to gain an unprecedented understanding of the photocatalytic hydrogen evolution reaction (HER) activity, of MIP‐177(Ti)‐LT and HT, and discuss these results in the context of their photocatalytic activity. We reveal that both MOFs show distinct transient IR signatures following pulsed excitation at a wavelength of 355 nm. Also, the transient changes in the MIP‐177(Ti)‐LT IR spectrum proceeds for a longer time than that of MIP‐177(Ti)‐HT, in correlation with transient absorption measurements in the UV–vis range.

## 2 Results

The prepared MIP‐177(Ti)‐LT and MIP‐177(Ti)‐HT were characterized by SEM (Figure S1, Supporting Information), PXRD (Figure S2, Supporting Information), and N_2_‐adsorption (Figure S3, Supporting Information). The morphology and the average dimension distribution were found to be in agreement with previously reported data,^[^ ^6^ ^]^ confirming successful synthesis. The BET specific surface areas were found to be about 700 m^2^ g^−1^ for both MIP‐177(Ti)‐HT and MIP‐177(Ti)‐LT.

MIP‐177(Ti) solids were further characterized by XPS (Figures S4 and S5, Supporting Information). In general, C1s XPS core level can be decomposed into three bands with binding energies at about 284.4 286.2 and 288.2 eV, associated to aromatic benzene ring carbons, ─CH_2_─ groups and the carboxylate moieties of the ligands, respectively. The broad O1s XPS peaks (526 to 536 eV) can be associated to oxygen in Ti─O bonds (≈530 eV) or present in carboxylates (≈532 eV). The XPS peaks of the Ti 2p regions are assigned to the presence of Ti(IV), as manifested by the two bands at 458.2 and 463.8 eV corresponding to Ti2p_3/2_ and Ti2p_1/2_, respectively. The HOCO of MIP‐177(Ti)‐HT and MIP‐177(Ti)‐LT (1.77 and 1.91 vs NHE, respectively) was calculated based on the Fermi levels of the MOFs, determined by XPS (Figure S6, Supporting Information), as described in the experimental section.

**Figure 2:**
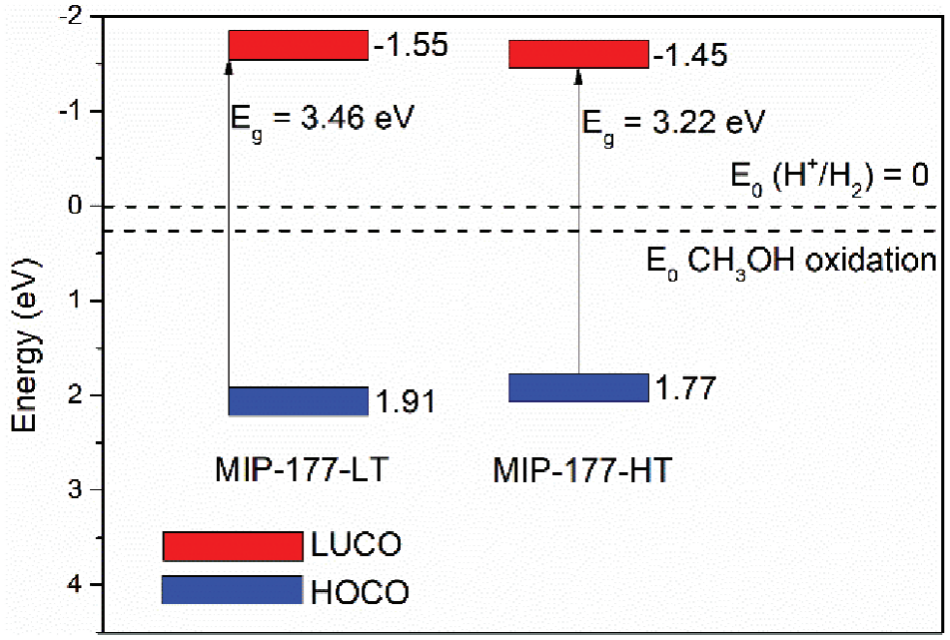
The band energy diagram of MIP‐177(Ti)‐LT and MIP‐177(Ti)‐HT as determined from XPS and UV‐Vis DRS measurements.

The optical properties of MIP‐177(Ti) samples were studied by UV–Vis diffuse reflectance spectroscopy (DRS). The spectra show a broad band from 200 to 300 nm, associated to the electronic transitions of ligand to metal charge transfer in O─Ti bonds (2p →3d), together with electronic transitions of the organic ligand (π→π*). From this UV–Vis absorption data and using the Tauc plot analysis (Figure S7, Supporting Information), the optical HOCO–LUCO gap of MIP‐177(Ti)‐HT and MIP‐177(Ti)‐LT were determined to be 3.22 and 3.46 eV, respectively. **Figure** **2** presents the overall band energy diagram of these two MOFs, based on the calculated HOCO─LUCO gaps and the HOCO energy levels. As portrayed in the figure, the thermodynamic potentials of the MOFs are sufficient for their use as photocatalysts for HER using methanol as a sacrificial electron donor.

### 2.1 Steady State IR Spectra

**Figure 3:**
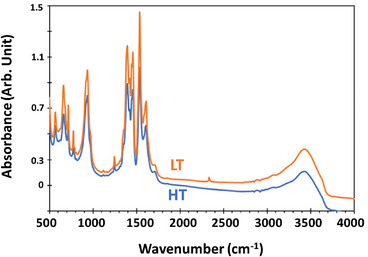
Steady state FTIR spectra of MIP‐177(Ti)‐LT (LT) and MIP‐177(Ti)‐HT (HT).

**Figure** **3** shows that the MID‐IR signal (500 cm^−1^ to 4000 cm^−1^) of MIP‐177(Ti)s is composed of two separate regions: The Ti─O stretching vibrations (500–800 cm^−1^) and the organic ligand vibrations between (800 to 4000 cm^−1^). Among the observed peaks, 509 cm^−1^ and 656 cm^−1^ represent two Ti─O─Ti stretching vibrations,^[^ ^14^ ^]^ while the benzene ring vibrations, the symmetric and asymmetric carboxylate stretching vibrations are found at 900–1000 cm^−1^, 1400–1410 cm^−1^ and 1550–1600 cm^−1^, respectively.^[^ ^15^ ^]^ The strong, very wide peak around 3400 cm^−1^, is associated with H bonded hydroxyl groups, belonging to carboxylate groups in the ligands and to adsorbed water. Close examination of the two spectra reveals that the IR spectrum of MIP‐177(Ti)‐LT is almost identical to that of MIP‐177(Ti)‐HT, despite the lack of bridging formates between the Ti─O clusters and the difference in the Oxo/Ti ratio (1.25 and 1.5 in the MIP‐177(Ti)‐LT and MIP‐177(Ti)‐HT, respectively).

### 2.2 Transient IR Signals of Pristine MOFs

**Figure 4:**
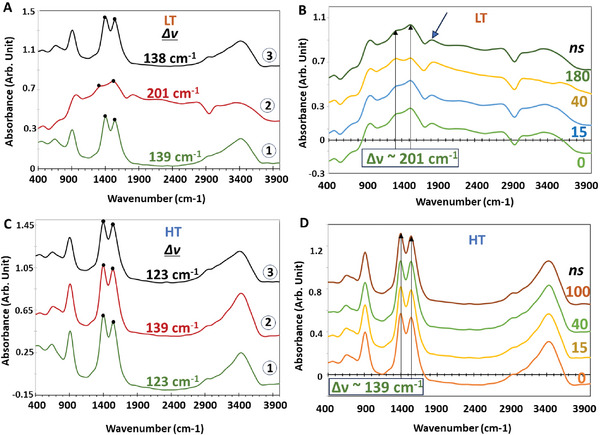
A) Spectra of MIP‐177(Ti)‐LT at *t* = 0, prior to laser exposure (1), *t* = 0, during the transient measurement (2), and following complete relaxation after 11 minutes of successive exposure to 355 nm pulses at 10 Hz (3). B) Transient spectra of MIP‐177(Ti)‐LT at different times (in ns) from excitation. C) Spectra of MIP‐177(Ti)‐HT at *t* = 0, prior to laser exposure (1), *t* = 0, during the transient measurement (2), and following complete relaxation after 11 min of successive exposure to 355 nm pulses at 10 Hz (3). D) Transient spectra of MIP‐177(Ti)‐HT at different times (in ns) from excitation.

**Figure** **4**A,C portrays the transient IR spectra of MIP‐177(Ti)‐LT and MIP‐177(Ti)‐HT, respectively, prior to laser exposure and following several laser shots at 355 nm pulses required for performing the transient measurements. Evidently, for both types of MOFs, it is impossible to observe any difference between the pre‐measurement spectra and the post‐measurement spectra. This suggests that exposure to the laser beam during the measurements does not cause any long‐term damage of the MOFs that can be detected by IR spectroscopy.

Figure 4B presents the evolution of the transient IR spectrum of MIP‐177(Ti)‐LT following excitation. The spectra obtained during the transient measurements substantially differ from the spectrum that were collected initially (Figure 3). Three main differences are observed: 1) an increase in absorption over very large ranges: from 1000 cm^−1^ to 1300 cm^−1^ and from 1900 cm^−1^ to 2900 cm^−1^ 2) the appearance of a new peak at 1850 cm^−1^ 3) changes in the spectral location of ν(COO^−^)_as_ and ν(COO^−^)_s_, from 1404 cm^−1^ to 1326 cm^−1^ and from 1542 cm^−1^ to 1527 cm^−1^, respectively. These changes were observed not only upon excitation, but, quite surprisingly, also at *t* = 0, i.e., at a time that apparently was prior to excitation

As described in the experimental part, the transient IR measurements were performed by FTIR, working in a “step‐scan” mode. In this mode, each spectrum associated with some time (*t*_i_) relative to excitation (including t_0_) is the outcome of a Fourier transformation of data recorded over a large number of excitation events. Since excitation took place at a frequency of 10 Hz, the spectrum at time zero, right before excitation, is actually the residual spectrum 100 ms from excitation. Hence, it can be deduced that although excitation does not cause a permanent change to MIP‐177(Ti)‐LT, it does generate spectral changes that persist longer than 0.1 second from excitation.

Similar to MIP‐177(Ti)‐LT, the MIP‐177(Ti)‐HT does not experience a permanent change upon excitation, as the corresponding transient IR spectra of this MOF hardly showed any spectral changes that persisted for 0.1 s, except for a minor change in the spectral location of ν(COO^−^)_as_ and ν(COO^−^)_s_, from 1558 cm^−1^ to 1542 cm^−1^ and from 1404 cm^−1^ to 1419 cm^−1^, respectively (see Figure 4C).

It is important to note that the energy difference, ∆ν (where ∆ν = ν(COO─)_as_ – ν(COO─)_s_), between these two carboxylate stretching modes is crucial for understanding the nature of metal–ligand coordination in the respective MOFs, both in the ground and in the excited states. A ∆ν value in the range of 180–210 cm⁻¹ typically indicates mono‐dentate coordination, while a value between 120 and 150 cm⁻¹ suggests bi‐dentate metal–ligand coordination.^[^ ^16^ ^]^ A comprehensive analysis of the varying ∆ν values observed in MIP‐177(Ti)‐LT and MIP‐177(Ti)‐HT, as illustrated in Figure 4A,C, respectively, is provided in the discussion section.

To gain insight into the different behaviors of MIP‐177(Ti)‐LT and MIP‐177(Ti)‐HT upon excitation and to understand how it impacts their photocatalytic properties one has to look in‐depth into the transient spectral changes right after excitation. These changes are better observed by analyzing the difference in the spectrum at some time after excitation to the spectrum measured just prior to excitation.

**Figure 5:**
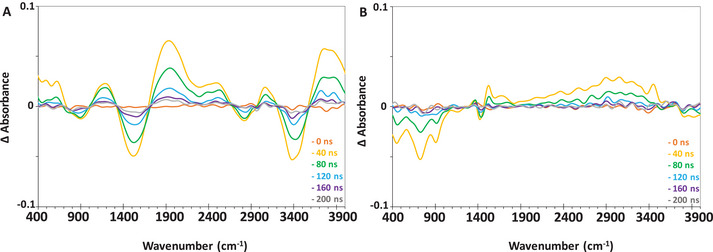
Transient difference spectra of A) MIP‐177(Ti)‐LT, and B) MIP‐177(Ti)‐HT following pulsed excitation at 355 nm.

**Figure** **5**A,B presents the transient difference spectra (Abs_t_‐Abs_0_) of MIP‐177(Ti)‐LT and HT obtained by subtracting the absorbance signal prior to excitation (actually the residual spectrum 100 milliseconds from excitation) from the transient absorbance signal. These changes were characterized by time‐dependent intensity changes. The magnitude of the spectral changes varied within several dozens of nanoseconds, and eventually vanished 200–300 ns from excitation. The nature and extent of the intensity changes, as well as their duration, was found to vary among the two different types of MIP 177 (Ti) versions, unlike the situation at steady state, in which the spectra of the two MOFs were practically identical.

For MIP‐177(Ti)‐LT, transient changes began to be observed 15 ns from excitation, manifested by an intensity increase at 509 cm^−1^, 656 cm^−1^, 1180 cm^−1^, 1800–2650 cm^−1^ (comprising of two partially overlapping envelopes at 1900 cm^−1^ and at 2400 cm^−1^), 3100 cm^−1^ and a broad envelope beginning at 3550 cm^−1^ and continuing beyond 4000 cm^−1^. Negative peaks in the difference spectra (i.e., transient decrease in absorption) were observed at 900 cm^−1^, 1400–1550 cm^−1^, 2800 cm^−1^ and 3200–3550 cm^−1^. The magnitude of both the positive and the negative intensity changes reached a maximum at 40–50 ns from excitation. The transient changes at 509 cm^−1^ and 656 cm^−1^, correspond to the Ti─O region. Figure S14 (Supporting Information) depicts the transient changes in specific peaks as a function of time. In comparison to the steady state measurements the positive signal over the whole Ti─O region represent a widening of the Ti─O related peaks. The Ti─O related peaks vanished much faster than that of the rest of the IR peaks (110–120 ns versus 170–180 ns from excitation, respectively). Some of the transient changes correlated with the specific functional groups of MIP‐177(Ti). Others, like the positive transient changes in the region of 1180 cm^−1^ and the complex behavior between 1800–3000 cm^−1^ had no parallel peaks in the steady state spectrum and could not be explained on the basis of intensity changes in steady‐state IR peaks.

The transient IR spectrum of MIP‐177(Ti)‐HT was found to be totally different from that of MIP‐177(Ti)‐LT. A decrease in intensity was observed at 500–800 cm^−1^, centered at 509 cm^−1^ and 656 cm^−1^; the exact wavenumber associated with the Ti─O─Ti vibration. A negative transient change was also found at 970 cm^−1^ and 1404 cm^−1^, assigned to benzene ring vibrations and symmetric vibrations of the carboxylate functional group, respectively. Positive transient changes were found circa 1556 cm^−1^, associated with the stretching of the carboxylate functional group in the ligand. In addition, a very wide positive signal (from 1900 cm^−1^ to 3400 cm^−1^), was observed, although there were no peaks between 1800 cm^−1^ to 3000 cm^−1^ in the steady state spectrum. Similar to the MIP‐177(Ti)‐LT, the transient changes in MIP‐177(Ti)‐HT began 15 ns from excitation and proceeded for 100–110 ns before complete relaxation, much faster than in MIP‐177(Ti)‐LT. Unlike in MIP‐177(Ti)‐LT, the relaxation time was quite identical in all spectral regions. The striking difference between the MIP‐177(Ti)‐HT and the MIP‐177(Ti)‐LT was the absence of the transient peaks at 1280 cm^−1^ (negative) and 1800 cm^−1^ (positive) observed in MIP‐177(Ti)‐LT.

#### 2.2.1 Transient IR Spectroscopy in the Presence of Charge Scavengers

In order to understand the origin of the transient signals, the pristine MIP‐177(Ti)‐HT and LT samples were pre‐treated with charge scavengers (AgNO_3_ as an electron scavenger and methanol as a hole scavenger) prior to measuring their transient spectra.

**Figure 6:**
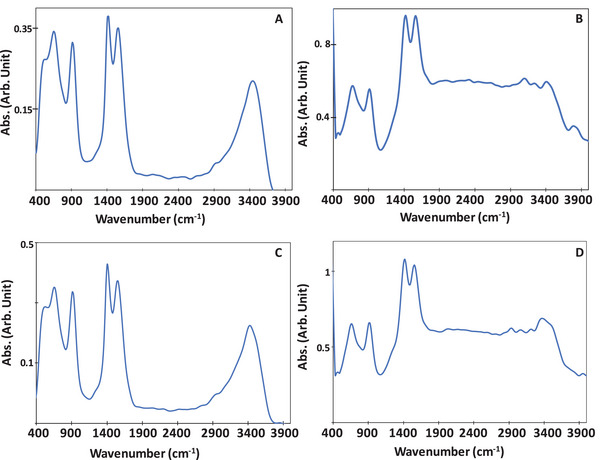
IR spectra of MIP‐177(Ti) 0.1 s from excitation (just before “new” excitation event) in the presence of charge scavengers. A) MIP‐177(Ti)‐LT with e^−^ scavenger, (B) MIP‐177(Ti)‐LT with h^+^ scavenger, C) MIP‐177(Ti)‐HT with e^−^ scavenger, and D) MIP‐177(Ti)‐HT with h^+^ scavenger.

**Figure** **6** presents the spectra at time zero during the step‐scan process, i.e., 0.1 s from excitation, just before a “new” excitation, for MIP‐177(Ti)‐LT (A, B) and MIP‐177(Ti)‐HT (C, D) in the presence of an electron scavenger (A, C) and hole scavenger (B, D).

In the presence of an electron scavenger, the spectrum of MIP‐177(Ti)‐HT was not only identical to that of MIP‐177(Ti)‐LT (Figure 6C vs A), but also identical to the pre‐excitation spectrum measured in the absence of a scavenger (Figure 4C‐2). In contrast, the spectrum of MIP‐177(Ti)‐LT in the presence of an electron scavenger at time zero during the transient measurements (Figure 6A) was found to be quite different from that measured in the absence of scavengers (Figure 4A‐2). With respect to MIP‐177(Ti) exposed to hole scavengers during excitation, there was very high similarity between the spectrum of MIP‐177(Ti)‐LT (Figure 6B) versus MIP‐177(Ti)‐HT (Figure 6D). Moreover, these two spectra showed high resemblance to the spectrum of MIP‐177(Ti)‐LT measured in the absence of scavengers (Figure 4A‐2) 0.1 s from excitation during the step scan process (i.e., right before “new” excitation). This similarity was manifested mostly by a very wide, feature‐less absorption band at 1900–3200 cm^−1^. It should be noted that transient measurements performed with KBr pellets exposed to methanol, in the absence of any MOF, did not yield any transient signal in the region of 600–4000 cm^−1^, let alone in the region of 1900–3200 cm^−1^ (Figure S7, Supporting Information), so that the long‐lasting transient signals at 1900–3200 cm^−1^, associated with H‐bonds, cannot be attributed to the methanol, but reflect transient phenomena in MOFs‐formed H‐bonds.

**Figure 7:**
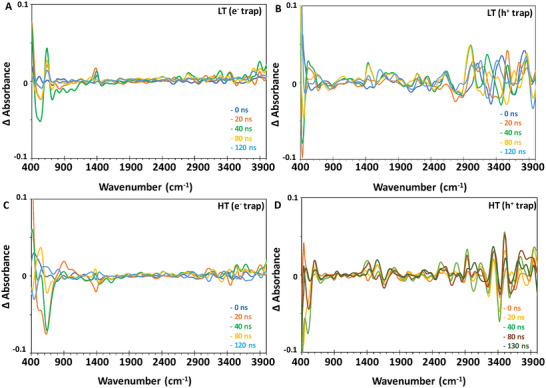
Transient IR difference spectra of MIP‐177(Ti) in the presence of charge scavengers. A) MIP‐177(Ti)‐LT (LT) with e^−^ scavenger, B) MIP‐177(Ti)‐LT with h^+^ scavenger, C) MIP‐177(Ti)‐HT (HT) with e^−^ scavenger, D) MIP‐177(Ti)‐HT with h^+^ scavenger.

The difference transient spectra with reference to the spectra at *t* = 0 (i.e., to spectra taken 0.1 s from excitation) in the presence of scavengers are depicted in **Figure** **7**. In the case of MIP‐177(Ti)‐LT, the addition of AgNO_3_ silenced most of the post‐excitation transient effects on the spectrum, in particular at wavenumbers larger than 1500 cm^−1^. Positive signals in the difference spectra were found at 630 cm^−1^ and at 1415 cm^−1^. A negative change in the absorption intensity was observed in the region of 700 cm^−1^ to 1150 cm^−1^. Post excitation transient changes at 630 cm^−1^ began 10 ns from excitation, reached their maximum intensity some 28–35 ns from excitation and completely vanished at 140–145 ns from excitation. The transient changes at 1415 cm^−1^ also started 7–10 ns from excitation and reached their maximum at 30–35 ns from excitation, unlike the transient signal at 630 cm^−1^, that vanished much faster, within 55–60 ns from excitation.

The most prominent change in the transient spectrum of MIP‐177(Ti)‐HT upon introducing electron scavengers (Figure 7C) was the disappearance of the positive Δabs signal at 1900–3500 cm^−1^. In addition, an increase in the magnitude of the negative Δabs at 640–660 cm^−1^ was found, together with the appearance of a positive Δabs at 950–1000 cm^−1^, a region that showed a negative Δabs signal in the pristine MIP‐177(Ti)‐HT (Figure 5B).

The transient spectra were altered in both MOFs not only in the presence of electron scavengers, but also in the presence of hole scavengers. Here, the most noticeable effect of the presence of MeOH was the appearance of a group of Δabs signals, some of which positive, and some negative, in the 3000–4000 cm^−1^ region. These signals, which were more pronounced in MIP‐177(Ti)‐LT than in MIP‐177(Ti)‐HT, persisted for a long time, up to 1400 ns from excitation. The wide envelope at 3000–4000 cm^−1^ is likely to reflect the presence of H bonds, originated from adjacent OH groups, which can be formed in different configurations with different number of neighbors,^[^ ^17^ ^]^ hence the observed behavior suggests re‐distribution of H‐bonding, caused and stabilized by the presence of electrons next to the groups that form the H bonds. Another effect, more pronounced in MIP‐177(Ti)‐LT, was a change of the 1420 cm^−1^ peak, from a negative peak (Figure 5) to a positive one (Figure 7), which, over time, became negative again, before disappearing.

**Figure 8:**
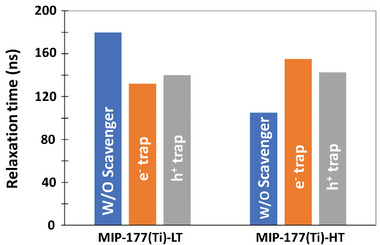
The relaxation time for MIP‐177(Ti)‐LT (left) and MIP‐177(Ti)‐HT (right), as measured between 600 cm^−1^ and 1600 cm^−1^. For each MOF, three conditions are shown: w/o a scavenger (blue), with e^−^ scavenger (orange) and with h^+^ scavenger (gray).

The effect of scavengers on the averaged relaxation time of the spectral signals between 600 cm^−1^ and 1600 cm^−1^ is given in **Figure** **8**. The data is presented only for that range, since this is the range of resolved localized vibrational transitions. As shown in Figure 4A‐1,C(1), the range between 1900 cm^−1^ and 2900 cm^−1^ has no distinct vibrational transitions (at least in the ground electronic state (Figure 3), but exhibited residual signal even after 0.1 s, hence it deserves a different treatment. Figure 8 clearly shows the difference between the MIP‐177(Ti)‐LT type to the MIP‐177(Ti)‐HT type. The introduction of scavengers (regardless of type) decreased the relaxation time for MIP‐177(Ti)‐LT, whereas for MIP‐177(Ti)‐HT, the relaxation time was increased. These observations are discussed in the discussion section.

Since, in general, the scavengers adsorb on the outer surface of a photocatalyst, a noticeable effect of scavengers on the transient signal of semiconductors may testify that the surface plays a dominant role (either directly or indirectly) in the transient spectral changes. The situation with MOFs can be quite different due to the inherent penetrability of these structures to small molecules and ions.

### 2.3 UV–Vis Transient Absorption Measurements

**Figure 9:**
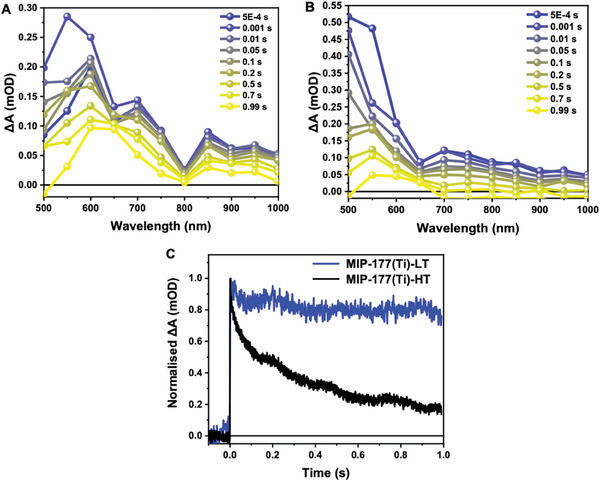
DR‐TAS spectra of A) MIP‐177(Ti)‐LT B) MIP‐177(Ti)‐HT measured on film in Ar, excited by a 355 nm laser with an intensity of 270 µJ cm^−2^ and a frequency of 1 Hz. C) Normalized DR‐TAS kinetics (DAQ) comparison of MIP‐177(Ti)‐LT and MIP‐177(Ti)‐HT, measured under the same conditions as above and probed at 650 nm.

Diffuse‐reflectance transient absorption spectroscopy (DR‐TAS) was employed to investigate the charge carrier decay behaviors in MIP‐177(Ti)‐LT and MIP‐177(Ti)‐HT in the solid state over microsecond to second timescales. The MOFs were excited using a 355 nm pulsed laser with an intensity of 270 µJ cm^−2^ and a frequency of 1 Hz. The absorption spectra of both MIP‐177(Ti)‐LT and MIP‐177(Ti)‐HT showed a positive photoinduced absorption signal of charge carriers in the 500–1000 nm range (**Figure** **9**A,B), with the main absorption peak occurring around 500–700 nm and decaying slowly over time, similar to the reported hole‐spectrum of TiO_2_.^[^ ^18^ ^]^ No ground‐state bleaching was observed in the measurement range, aligning with the steady‐state UV‐Vis absorption of these materials (Figure S7A, Supporting Information), which indicates absorption only in the UV range. Normalized kinetics at 650 nm reveal that the charge carriers in MIP‐177(Ti)‐LT decay much more slowly than those in MIP‐177(Ti)‐HT. This observation is consistent with the slower decay observed in MIP‐177(Ti)‐ LT compared to MIP‐177(Ti)‐HT in time‐resolved IR studies (Figure 9C).

### 2.4 Photocatalytic Activity Measurements and Photocurrent Response

The photocatalytic HER activity of MIP‐177(Ti)‐LT and MIP‐177(Ti)‐HT was studied under simulated sunlight irradiation. The obtained results show that the activity of MIP‐177(Ti)‐LT (about 6.8 mmol g^−1^ after 21 h of irradiation) is higher than that of MIP‐177(Ti)‐HT (about 4.5 mmol g^−1^ after 21 h of irradiation), i.e., the MIP‐177(Ti)‐LT is more efficient by a factor of 1.38 (or 1.26 upon considering Ti mass as the basis for comparison).

**Figure 10:**
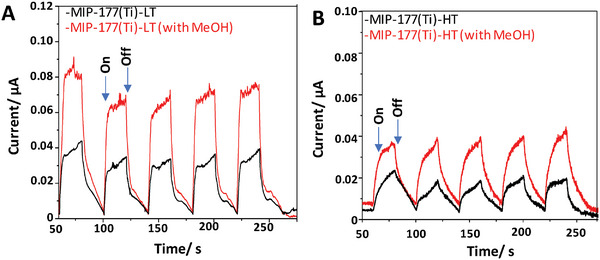
Transient photocurrent response of A) MIP‐177(Ti)‐LT and B) MIP‐177(Ti)‐HT supported on conductive carbon paper as the working electrode polarized at +0.9 V and submerged in an Ar purged acetonitrile solution of 0.1 m, TBAPF_6_ (black lines) or a mixture of acetonitrile and methanol (0.2 mL MeOH) solution during five consecutive on/off cycles under simulated sunlight irradiation.

The better performance of MIP‐177(Ti)‐LT relative to that of MIP‐177(Ti)‐HT was confirmed by transient photocurrent measurements under simulated sunlight irradiation and a bias of 0.9 V (**Figure** **10**). Analogous experiments performed in the presence of a hole scavenger (methanol) further increased the measured transient photocurrent for both MOFs. This observation is in accordance with the role of methanol as an electron donor, becoming oxidized by the photogenerated holes of the MOFs, partially avoiding electron/hole recombination. This, therefore, favors electron extraction from the electrodes and, thus, an increase in photocurrent intensity. Again, the higher transient photocurrent activity of MIP‐177(Ti)‐LT compared to MIP‐177(Ti)‐HT, indicating that more electrons can be extracted from this MOF, is in line with the higher HER activity of MIP‐177(Ti)‐LT. Furthermore, MIP‐177(Ti)‐LT also demonstrated reusability with minimal loss in activity after three reuse cycle (Figure S9A, Supporting Information). PXRD analysis of the spent catalyst following three reaction cycle (Figure S9B, Supporting Information) confirmed that the material retained its crystallinity throughout the reusing process.

The difference between the transient photocurrent behavior of MIP‐177(Ti)‐HT and that of MIP‐177(Ti)‐LT was not limited to the photocurrent values, but was manifested via their time dependent response to the on/off irradiation switching. Here, both the rise time constant (τ_r_) and the decay time constant (τ_d_) were higher for the MIP‐177(Ti)‐LT than for the MIP‐177(Ti)‐HT (0.19 vs 0.1, respectively for tr and 0.008 vs 0.083, respectively, for td). This difference indicates that MIP‐177(Ti)‐HT has a more resistive‐capacitive (RC) character than MIP‐177(Ti)‐LT. This is expected to be manifested by a reduced number of mobile charges available for the reaction.^[^ ^19^ ^]^

## 3 Discussion

Despite the apparent very high spectral resemblance in the IR region between MIP‐177(Ti)‐LT and MIP‐177(Ti)‐HT, measured in the ground state, our results showed that the apparent minor structural difference between MIP‐177(Ti)‐LT and MIP‐177(Ti)‐HT (polymerization of Ti─O clusters into inorganic chains) had a noticeable effect on the transient response of these MOFs to light exposure.

Probably the most striking difference is the lasting (>0.1 s), featureless increase in the absorption signal arriving from MIP‐177(Ti)‐LT, between 1800 cm^−1^ and 2900 cm^−1^, a spectral region which has no distinct vibrational transitions in the ground state (trace 1 in Figure 4A vs B). The lack of specific peaks in the spectrum in this area (except for an apparent peak at 2100 cm^−1^ in the MIP‐177(Ti)‐LT type) negates the possibility of specific vibrational IR transitions at the electronically excited state. Similar featureless increase in absorption signal at this range, however for shorter time, was found also with MIP‐177(Ti)‐HT. A possible explanation for the non‐specific IR absorption is the presence of an electronic, non‐localized, band within the HOCO–LUCO gap so that the IR absorption is not due to vibrational transitions but reflects electronic transitions. Indeed, similar featureless changes in IR absorption, explained by the presence of minibands, were found also in excited TiO_2_,^[^ ^20^, ^21^ ^]^ and in graphitic carbon nitride,^[^ ^22^ ^]^ and are the outcome of conduction band electrons/valence band holes. In terms of energy, the range at which the non‐specific IR absorption took place in both types of MIP‐177(Ti) was quite limited. For this reason, we refer to it not as a “band”, but, rather, as a “miniband” (mb).

**Figure 11:**
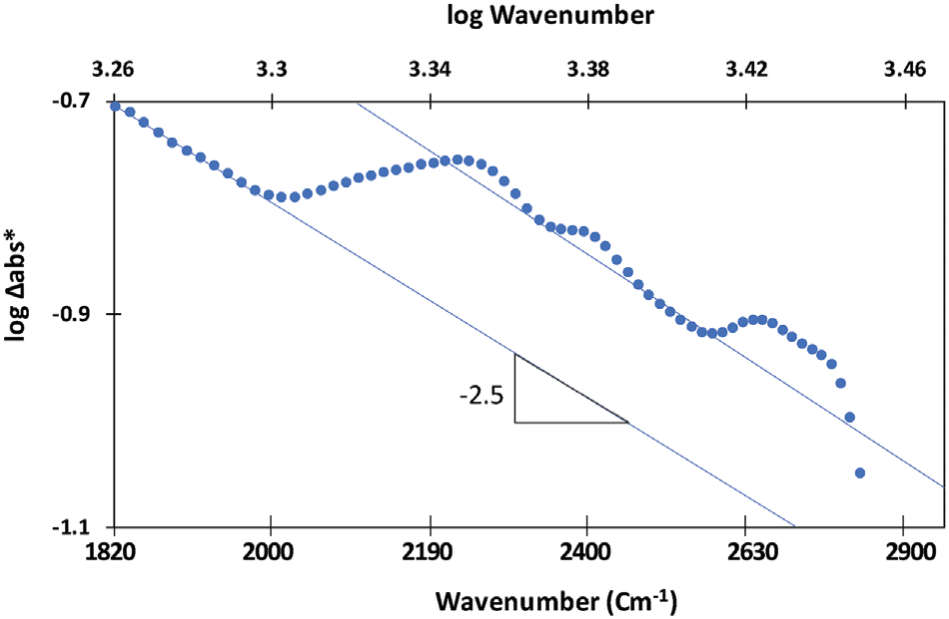
The correlation between log Δabs* and log wavenumber in the featureless absorption region (1800–2900 cm^−1^) in MIP‐177(Ti)‐LT.

The dependence of the long‐lived absorption in the 1000–1300 cm^−1^ and 1900–2900 cm^−1^ ranges was calculated by subtracting the absorption spectrum taken prior to the transient measurement from the *t* = 0 spectrum during the transient measurement (i.e., 0.1 s from excitation). This subtraction spectrum is denoted hereby as Δabs*. **Figure** **11** presents Δabs* versus wavenumber in a log–log scale for the two regions. A linear correlation between log Δabs* and log ν indicates a power law (Δabs*∝ν^−β^). In such cases, the value of β depends on the mechanism of absorption.^[^ ^22^ ^]^ As shown in Figure 11, the 1800–2900 cm^−1^ part of the spectrum is comprised of two regions having a linear dependence on log wavenumber with a slope of ‐2.5±0.2. In between, a region in which the slope is positive, is found. This intermediate region is located at 2000–2210 cm^−1^, coinciding with a specific peak found in the transient and in the long‐lasting spectra, so no wonder it masks the slope. It is known that a −*β* value of ‐2.5 is associated with electronic transitions involving optical phonons, whereas an order of −3.5 is associated with electronic transitions involving scattering from charged impurities,^[^ ^23^ ^]^ so it is reasonable that this is the case also here. Quite surprisingly, the ‐β value for the 1000–1300 cm^−1^, another region where a distinct difference was found between the long‐lasting spectrum and the pre‐excitation spectrum, was positive, hence cannot be explained by the involvement of phonons.

Having in mind that the non‐specific IR absorption was due to electronic transitions, one may try to locate the miniband within the energy scale. In doing so, one has to explain the presence of non‐specific signals in the two regions: 1000–1300 cm^−1^ and 1800–2900 cm^−1^ (it is possible that this region extends beyond 2900 cm^−1^, nevertheless, we limited it to 2900 cm^−1^ due to an overlap with the OH–related broad band above 3000 cm^−1^).

The miniband can be located either at the vicinity of the conduction band (option 1), or at the vicinity of the valence band (option 2). In option one, the miniband is not populated at steady state and becomes populated once excited electrons are relaxed from the conduction band to the miniband. In that case the IR absorption excites electrons that reside in the miniband either internally within the miniband or back to the conduction band. In option two, the process is less straight‐forward. Prior to excitation the miniband was populated (otherwise, if it was not populated, one could expect IR absorption also at steady state). The laser excitation drives electrons from the HOCO to the LUCO, while forming holes at the HOCO. Then, electrons from the miniband are relaxed to the HOCO, leaving empty states within the miniband. In that case, IR absorption is manifested by excitation of HOCO electrons, either internally within the miniband/HOCO or, alternatively, from the HOCO to the miniband.

Our results clearly showed that the presence of electron scavengers eliminated the transient signals recorded for MIP‐177(Ti)‐LT, not only at the long‐time scale (Figure 6A) but also at a short time domain (Figure 7A). In contrast, the presence of hole scavengers yielded long‐lasting absorption signals at 1900–3300 cm^−1^. These results support the notion that in MIP‐177(Ti)‐LT the miniband is located near the LUCO (option 1) thus negating option 2.

Considering that the miniband is located next to LUCO raises a following‐up question regarding the nature of the IR non‐specific absorption. Are these transitions within the miniband, or from the miniband to the LUCO? In answering these questions, one may need to explain why was the non‐specific absorption observed only between 1000 cm^−1^ to 1300 cm^−1^ and 1900 cm^−1^ to 3200 cm^−1^? Moreover, why one does not observe this type of IR absorption circa 3600–4000 cm^−1^ (Figure 4B)?

**Figure 12:**
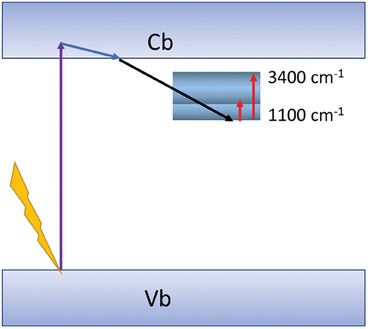
A schematic representation of the proposed scenario involving non‐specific transient IR absorption at distinct regions. Laser excitation, intra‐band relaxation, trapping in the miniband and IR absorption are represented by velvet, blue, black and red arrows, respectively.

This enigma may be explained by the presence of a miniband having, within it, distinct regions of high density of states, as portrayed in **Figure** **12**. The non‐specific, electronic, IR transitions take place within this miniband from the lower region of high density of states to the other two regions of high density of states. The first some 1100 cm^−1^ from the bottom of the miniband and the second approximately 2500 cm^−1^ from this bottom. Such a configuration, in which the IR excitation takes place within the miniband and not to the LUCO explains the disappearance of signal close to 4000 cm^−1^.

It should be noted that this type of explanation is depicted in terms of band theory which is more appropriate for systems having strong coupling between all atoms. The situation in MOFs is less strict, which means that the edges of the bands are relatively “soft”.

For MIP‐177(Ti)‐HT, the spectrum measured 0.1 s from excitation in the presence of a hole scavenger (Figure 6D) and in the presence of an electron scavenger (Figure 6C) indicates the presence of a miniband which is quite similar to that of MIP‐177(Ti)‐LT. Nevertheless, from Figure 4D it is evident that in the absence of a scavenger this miniband is not populated 0.1 s from excitation, indicating fast recombination in the MIP‐177(Ti)‐HT type. The faster recombination of MIP‐177(Ti)‐HT relative to that of MIP‐177(Ti)‐LT can be inferred also from the diffuse reflectance transient absorption results (Figure 9).

In both MIP‐177(Ti)‐LT and MIP‐177(Ti)‐HT the carboxylate group of the methylene di‐isophthalate (mdip) ligand is chelated to the Ti─O cluster. As mentioned in the previous section, the energy difference between the asymmetric transition and the symmetric transition of carboxylate ligands (∆ν) is indicative of the coordination of the carboxylate.^[^ ^16^ ^]^ The ∆ν of 130 cm^−1^ measured for both MIP‐177(Ti)‐LT and MIP‐177(Ti)‐HT, indicated a bidentate coordination in the absence of excitation. As shown in Figure 4, the position of the ν(COO^−^)_as_ and ν(COO^−^)_s_ in MIP‐177(Ti)‐LT was altered upon excitation. This change was found to be quite long‐lasting (>0.1 s), nevertheless, reversible. It is manifested by an increase in ∆ν from 138 to 201 cm^−1^, that can be regarded as indicative for a temporal change from the bidentate coordination to a monodentate coordination. This change in coordination was not found in MIP‐177(Ti)‐HT, although both types share the same mdip functional group.

It should be noted that transient measurements performed with charge scavengers indicated the presence of a bidentate interaction between the mdip group and the Ti‐O cluster regardless of scavenger type. Hence, it can be concluded that both types of carriers are instrumental for the transformation from the bidentate state to the monodentate state.

**Figure 13:**
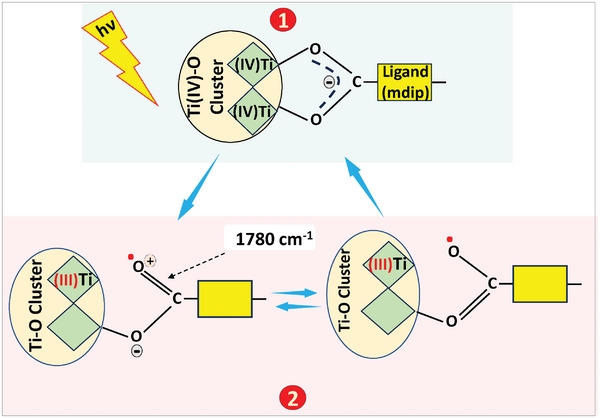
Schematics of the photoinduced coordination change phenomenon in MIP‐177(Ti)‐LT. (1) depicts bidentate coordination at ground state, while (2) shows the monodentate coordination at excited state.

A correlation was found between the three long‐lasting changes in the IR spectrum in MIP‐177(Ti)‐LT upon excitation: the electronic population of a miniband below Ec, the transformation of the mdip functional group (carboxylate) from a bidentate coordination to a monodentate coordination and the appearance of a new peak at 1780 cm^−1^. This peak can be ascribed to C═O,^[^ ^24^ ^]^ hence its presence contributes to our conclusion regarding the bidentate to monodentate transformation. The schematics of this dynamic change is depicted in **Figure** **13**. Similar dynamic transformation from a bidentate coordination to a monodentate coordination was recently reported in another MOF (MIL‐101(Fe)),^[^ ^25^ ^]^ so apparently this transformation may reflect a feature that is common to many similar MOFs.

In MIP‐177(Ti)‐LT, the transient changes in the Ti─O region were manifested by an apparent broadening of the absorption envelope, as can be inferred from Figure 5. This widening may represent inhomogeneous broadening, reflecting an increase in the heterogeneity of the Ti─O bonds. What causes this heterogeneous broadening is likely to be the effect of a near‐by trapped charge, whose effect of the Ti─O potential varies as a function of distance, thus affecting each Ti─O bond in a different manner.

Upon introducing an electron scavenger, one observed a positive ∆abs peak at 630 cm^−1^, located between two negative ∆abs peaks (at 540 cm^−1^ and 709 cm^−1^). This behavior is typical for narrowing, (i.e less inhomogeneous broadening) caused by depleting the electrons from the Ti─O clusters. The asymmetry between the strong effect of the electron scavenger and the milder effect of the hole scavenger suggests that the trapped charge in the Ti─O cluster is negative, transforming Ti(IV) into Ti(III).

As shown in Figure 8, scavengers increased the post excitation relaxation time of the transient signal for MIP‐177(Ti)‐HT, but decreased it for MIP‐177(Ti)‐LT. Explaining the observation for the former is rather intuitive: prevention of a recombination process due to removal of counter charges. The observation for MIP‐177(Ti)‐LT is counter‐intuitive, hence may represent a more complicated explanation. It is proposed that this observation is connected with the prevention of bi‐dentate to mono‐dentate transformation by the scavengers (as can be deduced from Figures 6 and 7). Since monodentate configuration plays an important role in charge transport away from the excitation loci, the outcome could be localization of part of the electron–hole couples within a limited space, thus increasing the probability of recombination.

All this bring to the conclusion that the excited electrons survive for quite a long time (>0.1 s) while being located on the separated Ti─O clusters, and, from an energetic point of view, at a miniband located below Ec. Indeed, the long‐lasting effect (Figure 4B) is not limited to the 1900–2900 cm^−1^ region, but, in fact, is manifested by a reduction in the absorption of the Ti─O envelope, as one may infer from comparing Figure 4B with A. This reduction in intensity can be explained by lower polarizability, reflected in a decrease of absorption coefficient.

Hints on the whereabout of holes can be found by observing the effect of scavengers on the IR signal of the ligands, in particular that of the benzene ring at ca. 900–1000 cm^−1^. Here, the presence of an electron scavenger hardly affected the transient signal, while the presence of holes eliminated the transient signal at this region. This suggests that the transient signal in this region was the outcome of the presence of holes.

Overall, our results indicate the presence of holes in the ligand and the presence of electrons in the Ti─O cluster. While the presence of electrons scavengers is very dominant, similar effect was found with hole scavengers, although much weaker. In particular, the transformation of mdip carboxylate from a bidentate coordination to the Ti─O cluster to a monodentate coordination requires both types of charges. This may indicate that the two opposite charges are located not too far from each other. The MIP‐177(Ti)‐LT structure, comprising from separated Ti─O clusters contributes to this situation, as it is unfavorable for the electrons to move from one Ti─O cluster to the neighboring.

As discussed above, similar to MIP‐177(Ti)‐LT, the electronic structure of the MIP‐177(Ti)‐HT type has a miniband below Ec, however this miniband does not accommodate electrons in a long‐lasting manner. This is deduced from the presence of a featureless increase in absorption in the 1800–3000 cm^−1^ region that disappears within 100–200 ns, hence is not reflected in the *t* = 0 transient spectrum of the MIP‐177(Ti)‐HT type (Figure 4D). The lack of a bidentate‐to‐monodentate transformation, which, as shown for the MIP‐177(Ti)‐LT, requires both types of charges, implies that the electrons and holes in the MIP‐177(Ti)‐HT structure do not reside in a close proximity.

One possibility for a separation between electrons and holes is electron transport along the 1D Ti─O chains in the MIP‐177(Ti)‐HT. However, in this case, one could expect a slower recombination rate for the MIP‐177(Ti)‐HT type, quite in contrast to our observation.

The strong signals observed for both MIP‐177(Ti)‐LT and MIP‐177(Ti)‐HT in the presence of hole scavengers at 3200–4000 cm^−1^, suggest that electrons may reside in loci rich in OH groups responsible for H‐bonds. Such loci can be adsorbed water, which are likely to be on the Ti─O sub‐units, that exist in both types of MOFs. According to this explanation, the polymerization of the Ti─O subunits following heat treatment does not improve charge transport, simply because the photo induced electrons remained on water adsorbed on “their” Ti─O cluster. Another possibility is the difference between the Ti_6_O_9_ clusters of the MIP‐177(Ti)‐HT phase and the Ti_12_O_15_ clusters of the MIP‐177(Ti)‐LT structure, which may affect delocalization (see below, DFT results). Regardless of the exact reason, the longer lifetime of the excited state in MIP‐177(Ti)‐LT is supported also by the transient photocurrent measurements (Figure 10), revealing larger RC in MIP‐177(Ti)‐HT than in MIP‐177(Ti)‐LT.

This explanation rationalizes why we did not observe a slower recombination for the MIP‐177(Ti)‐HT type, but it cannot explain the significantly faster recombination for this type in comparison with the MIP‐177(Ti)‐LT type. Such a faster recombination could occur, if, for some reason, both carriers in MIP‐177(Ti)‐HT reside on the inorganic part. Two observations seem to support such a notion. First, the lack of bidentate to monodentate transformation, assigned to holes in organic part, in close vicinity to electrons in the inorganic part, indicates that the holes in MIP‐177(Ti)‐HT are located in places that are different from those in the MIP‐177(Ti)‐LT. Second, the transient signal at the Ti─O region of the spectra in the presence of electrons scavengers is quite different in the MIP‐177(Ti)‐HT from that of the MIP‐177(Ti)‐LT. Here, a strong intensity decrease at 656 cm^−1^ (O─Ti─O) was observed for the MIP‐177(Ti)‐HT type, indicating that this bond is altered by the presence of holes. Having both carriers in close proximity on the same Ti─O subunit can be the reason of a fast recombination (or, at least, faster than that of the MIP‐177(Ti)‐LT type).

**Figure 14:**
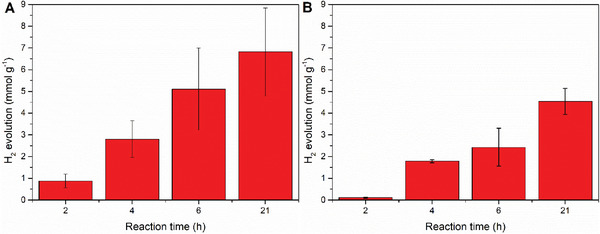
Photocatalytic HER using A) MIP‐177(Ti)‐LT and B) MIP‐177(Ti)‐HT as catalysts under simulated sunlight irradiation. Reaction conditions: Photocatalyst (5 mg), H_2_O (8 mL), MeOH (2 mL), sunlight irradiation power (300 mW cm^−2^).

It is, therefore, reasonable to assume that the slower recombination rate in MIP‐177(Ti)‐LT in comparison to that of MIP‐177(Ti)‐HT, is manifested by higher HER photocatalytic activity, as indeed measured by us (**Figure** **14**). This finding is, at first glance, surprising, since one could expect that polymerization of Ti─O clusters, would ease charge separation due to loss of confinement. Our results show beyond any doubt that this is not the case here.

**Figure 15:**
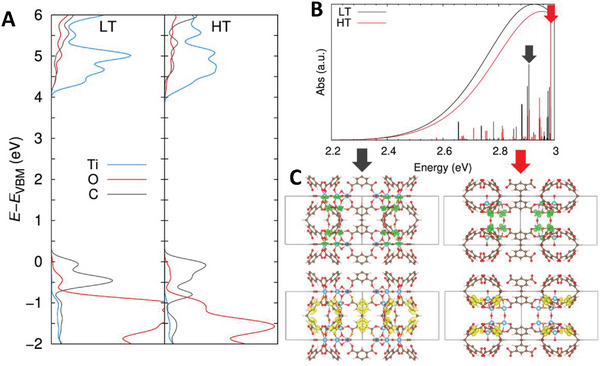
A) Projected density of states (PDOS) of the atoms conforming the MIP‐177(Ti)‐LT (left) and MIP‐177(Ti)‐HT structures, as calculated by employing hybrid functionals, where the valence band maximum (VBM) energy was set as reference. B) Simulated band edge absorption spectra for MIP‐177(Ti)‐LT (black) and MIP‐177(Ti)‐HT (red), where vertical bars represent the oscillator strengths of the states convoluting the spectra. The excited state properties of the main states belonging to this energy region are collected in Table S1 (Supporting Information). C) Lateral views of the shapes for the occupied (yellow) and virtual (green) crystalline orbitals involved in the main transitions of the excited states depicted with black and red arrows in the simulated spectra of MIP‐177(Ti)‐LT and MIP‐177(Ti)‐HT systems, respectively. Both top and lateral views for the rest of crystalline orbital transitions composing the states collected in Table S1 (Supporting Information) are depicted in Figure S11 (Supporting Information). The iso‐value used to plot the iso‐surfaces was set to 0.03 a.u.

With the aim of rationalizing the different recombination processes followed by the two versions of MIP‐177(Ti) we first computed their electronic structures by means of DFT calculations. **Figure** **15**A displays the projected density of states (PDOS) features for both phases. In line with our previous findings,^[^ ^6^ ^]^ valence band edge is composed by the C ligand states whereas the conductance band is mainly formed by the Ti states from the inorganic nodes; and hence, both compounds should exhibit, in principle, a similar ligand‐to‐metal charge transfer (LMCT) behavior. However, this picture is provided from a ground state perspective. In order to get a deeper understanding on their band edge absorption characteristics, TD‐DFT calculations were carried out to have access to the excited state properties of the lowest energy absorption region. Notably, MIP‐177(Ti)‐LT phase displays a slightly red‐shifted absorption with respect to the MIP‐177(Ti)‐HT one as it can be seen in the simulated absorption spectra displayed in Figure 15B, thus following a similar trend as the one observed in the Tauc plot spectra (Figure S7B, Supporting Information). We further analyzed the nature of the most optically active states present in this region by displaying the most relevant crystalline orbital transitions contributing to these states, which are represented in Figure 15C. Strikingly, despite their relatively close PDOS and absorption features, the distributions of the generated charges in MIP‐177(Ti)‐LT and MIP‐177(Ti)‐HT are relatively different. The generated holes in the MIP‐177(Ti)‐HT structure is mostly delocalized along the normal plane, whereas for MIP‐177(Ti)‐LT they are also distributed along the stacking direction. Interestingly, while the electron particles are highly confined in the boundaries of the Ti_6_O_9_ clusters for the MIP‐177(Ti)‐HT phase, they are fully delocalized along the Ti_12_O_15_ clusters for the MIP‐177(Ti)‐LT structure. Therefore, despite the expectation for a better electron transport along the 1D chain direction in the case of the MIP‐177(Ti)‐HT phase due to a continuous stacking of Ti_6_O_9_ moieties without any organic spacer (Figure 1), the breaking of the electronic communication between Ti‐O clusters hinders the paths for the electron migration, thus yielding to a fast recombination of the photogenerated charges, as it has been revealed above by time‐resolved spectroscopy measurements. Finally, with the objective of verifying the distinct delocalization paths observed for MIP‐177(Ti)‐LT versus MIP‐177(Ti)‐HT structures, we carried the natural transition orbital (NTOs) analysis, which takes into account all the crystalline orbital contributions for the excited states of interest (Figure S12, Supporting Information). Interestingly, despite the higher delocalization of the electrons in the NTO picture, a similar 3D versus 2D charge distribution was also found for MIP‐177(Ti)‐LT versus MIP‐177(Ti)‐HT phases within this type of analysis. Overall, the dielectric confinement of the photoinduced excitation of the MIP‐177(Ti)‐HT phase enhances the e–h interaction strength, thus hampering the separation of the photogenerated carriers and leading to a faster recombination. Analogous findings were observed when decreasing the dimensionality of other families of semiconductors.^[^ ^26^ ^]^

In a subsequent step, we elucidated the nature of the recombination processes in the two MIP‐177(Ti) forms by computing the radiative recombination lifetimes, as obtained from the transition probabilities provided by the TD‐DFPT. The lifetimes corresponding to the first ten excited states, (Table S2, Supporting Information) were estimated following the Kasha principle,^[^ ^27^ ^]^ according to which internal conversion radiative recombination occurs through the lowest energy states. Interestingly, all the computed lifetimes fall in the range from the few µs to the s timescale for both MIP‐177(Ti)‐LT and MIP‐177(Ti)‐HT. Furthermore, by applying again the Kasha principle^[^ ^37^ ^]^ the radiative recombination occurs through one of the three degenerated first excited states, which exhibited lifetimes in the second timescale. Consequently, radiative recombination should take place in timescales which are several orders of magnitude higher with respect to the lifetimes reported by transient‐IR measurements. Therefore, we can conclude that non‐radiative phenomena dominate the charge recombination processes in both MIP‐177(Ti)‐LT and MIP‐177(Ti)‐HT, in line with the findings already reported for other MOFs.^[^ ^28^ ^]^ Then, once we discarded the presence of band‐to‐band radiative recombination, we evaluated the suitability of both MIP‐177(Ti) forms to adopt non‐radiative processes driven by the bond breaking of the anchoring carboxylic groups (see Figure 13), as suggested by transient‐IR measurements. To this end, we estimated the potential energy surfaces (PES) of both ground and excited states corresponding to the Ti(cluster)─O(carboxylate) bond elongation from the equilibrium distance. The equilibrium Ti─O bond lengths of 2.0 and 2.1Å are, for the bonds formed with the O atoms, displaying larger and shorter carboxylic group C─O lengths (1.28 and 1.27Å, respectively) labelled as B1 and B2 (see Figure S13a, Supporting Information). Interestingly, these bonds exhibit similar ground state PESs for both MIP‐177(Ti)‐LT and MIP‐177(Ti)‐HT, going through a destabilization of about 0.7 and 0.5‐0.6 eV for B1 and B2, respectively, upon 0.5 Å of bond elongation (see Figure S13b, Supporting Information). More importantly, the excited state PES curves were found flatter, thus evidencing that the bond breaking process should take place upon light irradiation, as following the scheme represented in Figure 13. Interestingly, in contrast to the ground state PES, these excited PES curves differ between MIP‐177(Ti)‐HT and MIP‐177(Ti)‐LT. Typically, the destabilization of B1 is lower for MIP‐177(Ti)‐LT as compared to MIP‐177(Ti)‐HT (0.39 and 0.56 eV for MIP‐177(Ti)‐LT and MIP‐177(Ti)‐HT, respectively at Δ*d* = 0.5 Å), whereas B2 follows an opposite trend, MIP‐177(Ti)‐HT exhibiting the lower destabilization (0.43 and 0.29 eV for MIP‐177(Ti)‐LT and MIP‐177(Ti)‐HT, respectively at Δd = 0.5 Å). Thus, the mechanisms driving the formation of the monodentate binding upon light irradiation are different for MIP‐177(Ti)‐LT versus MIP‐177(Ti)‐HT phases. This is in line with the fact that this type of binding only exists for MIP‐177(Ti)‐LT.

## 4 Conclusion

A comparison was made between two types of MIP177(Ti). The first type, prepared at low temperature (MIP‐177(Ti)‐LT), comprised of Ti_12_O_15_ units, interconnected by terminal formate groups as well as by 3,3′,5,5′‐tetracarboxydiphenylmethane (mdip) moieties. The second type, (MIP‐177(Ti)‐HT) formed by a thermal process causing the rearrangement of the inorganic units, leading to 1D chain of Ti_6_O_9_ units, still coordinated to their mdip ligands. Based on the 1‐D structure of inorganic part it could have been expected a reduced rate of recombination in the MIP‐177(Ti)‐HT form and, consecutively, also higher activity. Nevertheless, HER photoactivity measurements clearly indicate that in fact MIP‐177(Ti)‐LT is much more active than MIP‐177(Ti)‐HT.

Combining transient IR measurements, UV–Vis transient absorption and DFT/TD‐DFPT calculations unveils the reasons for this situation. Both spectroscopic transient techniques, as well as photocurrent measurements, indicate that the average lifetime of the carriers is longer in the MIP‐177(Ti)‐LT type than in the MIP‐177(Ti)‐HT. The transient IR measurements indicate that the photoinduced electrons are located in the inorganic part while the holes are located in the mdip ligand. The difference between the MIP‐177(Ti)‐LT and the MIP‐177(Ti)‐HT versions is manifested in the transient IR transitions assigned to both the inorganic building units and the organic ligands. Here, the longer lifetime of the MIP‐177(Ti)‐LT is mapped onto a slower decay of the Ti‐O related peaks. Likewise, for the MIP‐177(Ti)‐LT, but not for the MIP‐177(Ti)‐HT, a reversible change in the coordination of the carboxylate groups from a bidentate coordination into a monodentate coordination was observed. Complementary DFT simulations show higher electron delocalization (hence, higher mobility) on the inorganic part of MIP‐177(Ti)‐LT than in MIP‐177(Ti)‐HT, thus explaining the superior catalytic activity for HER of the former over the latter. All in all, this work demonstrates that sophisticated transient spectroscopy techniques may be combined synergistically with DFT calculations in order to elucidate charge mobility not only in the ground state but, more importantly, also in the excited state.

## 5 Experimental Section

### Synthesis of MIP‐177(Ti)‐LT and MIP‐177(Ti)‐HT

The MIP‐177(Ti)‐LT was synthesized following the reported procedure by Wang et al.,^[^ ^6^ ^]^ where Ti(*i*PrO)_4_ and di(isophthalyl)methane (H_4_mdip) are mixed and reacted in formic acid under reflux conditions during 3 d. The MIP‐177(Ti)‐HT is obtained by heating the MIP‐177(Ti)‐LT in oven at 553K under ambient conditions during 12 h. Both samples were characterized by means of powder X‐ray diffraction, nitrogen sorption, FTIR and scanning electron microscopy (Supporting Information). The obtained data are in agreement with the previously reported ones.^[^ ^6^ ^]^

### Characterization Methods

Powder X‐ray diffraction (PXRD) data were collected using a high‐throughput Bruker D8 Advance diffractometer working on transmission mode and fitted with a focusing Göbel mirror. The X‐ray source was Cu‐Kα radiation (λ = 1.5418 Å). Nitrogen sorption isotherms at 77K were collected on a Micromeritics Tristar instrument equipped with LN_2_ bath. Prior to the adsorption measurement, the sample was degassed under vacuum (10^−3^ mbar) at 473K overnight using a Micromeritics VacPrep degassing unit. UV–Vis diffuse reflectance spectroscopy (DRS) measurements of MIP‐177 samples were carried out on a Varian spectrometer model Cary 5000 using a praying mantis accessory and BaSO_4_ as a standard for total reflectance.

X‐ray photoelectron spectra (XPS) of MIP‐177(Ti)‐LT and MIP‐177(Ti)‐HT were acquired on a SPECS spectrometer equipped with a hemispherical Phoibos 150–9MCD detector, using a monochromatic Al X‐ray source (K_α_ = 1486.6 eV). The C 1s peak at 284.4 eV was employed as reference for binding energy calibration. The Highest Occupied Crystal Orbital (HOCO) maximum values of these solids with respect to the Fermi level (E^f^_υ_) were also estimated by XPS. The HOCO band positions with respect to NHE (E_υ_^NHE^) were determined from the following equation: E_υ_^NHE^ = E_υ_^f^ + *ϕ*_sp_ + E_0_^NHE^, where *ϕ*_sp_ is the work function of the spectrometer used for the measurements (4.244 eV) and E_0_^NHE^ is the energy value of the NHE with respect to vacuum level of the electron (−4.44 eV). The LUCO band energy minimum value for each solid was calculated subtracting the optical bandgap from the calculated E_υ_^NHE^.

Transient photocurrent measurements were carried out using a Gamry Instruments potentiostat (model Interface 5000E), using a standard three‐electrode configuration. A platinum wire was employed as counter electrode and a saturated Ag/AgCl electrode as the reference electrode. The working electrodes were prepared using a paste made by dispersing MIP‐177(Ti) powder (15 mg) in a mixture of terpineol and acetone (0.18 mL and 0.6 mL, respectively), that was heated at 363 K for 12 h. Subsequently, the suspension was cooled down and 10 µL of it was spread onto an area of 1.0 × 0.5 cm^2^ on a conductive carbon Toray paper (2.0 × 1.0 cm^2^ size) using doctor blade technique. Afterwards, the electrode was treated at 423K for 12 h in air. The generated transient photocurrent intensity was measured by immersing the as‐prepared MIP‐177(Ti)‐based electrode in an electrolyte made of 0.1 m tetrabutylammonium hexafluorophosphate (TBAPF_6_) in Ar‐purged acetonitrile and polarizing at +0.9 V under simulated sunlight irradiation (LC‐8 light guide lamp; 1.5 AM filter, 300 mW cm^−2^). In the experiments, holes were quenched with methanol (0.2 mL) added to the acetonitrile solution.

### UV–Vis Transient Absorption Measurements

Samples were prepared by suspending 3.2 mg MOF in 500 µL of acetonitrile and sonicating for 10 min. 200 µL of the suspension was drop‐casted onto a clean glass measuring 1.5 × 1 cm, and the solvent was evaporated at room temperature. The glass was then placed into a 20 cm quartz cuvette (light path 1 cm), degassed with Ar for 30 min, and sealed with a rubber septum and parafilm.

A homebuilt diffuse‐reflectance transient absorption spectroscopy (DR‐TAS) setup was utilized to investigate charge carrier dynamics over microsecond to second timescales. Measurements on the MIP‐177(Ti)‐LT and MIP‐177(Ti)‐HT film samples were performed in the transmission mode with a 15% transmittance intensity filter. The laser source was generated by OPOTEK Opolette 355 II with a 7 ns pulse width. Data were recorded using a Tektronics TDS 2012B oscilloscope for microsecond to millisecond timescales, and a National Instruments NI USB‐6211 DAQ for millisecond to second timescales. Probe light was generated by a 100 W tungsten lamp (Bentham IL1) powered by a Bentham 605 power supply. Samples were excited at 355 nm with laser intensity of 270 µJ cm^−2^ and a laser frequency of 1 Hz. Probed wavelengths ranged from 500 to 1000 nm, with an average of 40 measurements per data point.

### Steady State and Time‐Resolved IR Measurements

Steady state FTIR measurements at room temperature were performed on a Bruker V70 spectrophotometer equipped with a DTGS detector and a temperature‐controlled holder, in vacuum. The same apparatus, coupled to an Nd:YAG pulsed laser (Q‐smart 450, 5 ns FWHM pulse, Quantel, UK), operating at 10 Hz, was used for transient IR measurements, performed by a step‐scan approach.^[^ ^4^ ^]^ Due to the step‐scan nature of the measurements and to the 10 Hz repetition rate of the laser, measurements taken at time zero might include some residual contribution from the previous excitation event, that occurred 100 milliseconds before. For excitation, the third harmonic (355 nm) of the laser was used. A fast and spectrally extended mercury cadmium telluride (MCT) detector was used, enabling to measure as of 550 cm^−1^ with a temporal resolution of 2.5 ns. The measured signal was stored and averaged over 20 pulses at each mirror position. Results were recorded at an intensity of 10 mJ per pulse by introducing appropriate attenuating filters (Kopp 5860 and 5840 filters). The whole IR beams path was under vacuum, to improve the signal to noise ratio. Samples were prepared by pressing a mixture comprised of 2.1 mg of KBr crystals, containing evenly‐spread 0.25% MOF, at 5 tons for 60 minutes yielding a homogeneous pellet. More details on the set‐up can be found elsewhere.^[^ ^29^, ^30^ ^]^

### Theoretical Methodology

Both MIP‐177(Ti)‐LT and MIP‐177(Ti)‐HT were computationally explored at the periodic density functional theory (DFT) level. The DFT‐geometry was adopted optimized hexagonal unit cells reported in the previous work,^[^ ^6^ ^]^ which correspond to cell parameters of *a* = *b* = 22.595Å and *c* = 12.307 Å for MIP‐177(Ti)‐LT, and of *a* = *b* = 21.816 Å and *c* = 11.985 Å for MIP‐177(Ti)‐HT. The ground state properties were computed within the hybrid Perdew‐Burke‐Ernzerhof functional (PBE0) employing an energy cut‐off of 600 Ry for the electron density. A double‐zeta‐valence‐polarized (DZVP) MOLOPT basis set^[^ ^31^ ^]^ was relied to represent valence electrons, while Goedecker−Teter−Hutter (GTH) pseudopotentials^[^ ^32^ ^]^ were employed for core electrons. Van der Waals interactions were accounted for by means of the Grimme's D3 method.^[^ ^33^ ^]^ Vertical excitations were estimated using the time‐dependent density functional perturbation theory (TD‐DFPT) linear response method,^[^ ^34^ ^]^ following the methodology employed in previous works assessing the excited state properties of MOF materials.^[^ ^35^, ^36^ ^]^ Within this approach TD‐DFPT calculations were carried out with the PBE functional, whereas a 200 Ry cut‐off and a convergence energy threshold of 10^−5^ eV were utilized. The use of pure DFT functionals, which usually underestimates the band gaps of semiconductors, is justified by the large size of the investigated cells, and the fact that the nature of the band edges is preserved when moving from the standard to the hybrid functionals, as it can be observed in Figure S10 (Supporting Information). The simulated absorption spectra were estimated by computing the first 200 lowest energy excited states and by gaussian convolution of the vertical transitions within a full‐width‐half‐maximum of *σ* = 0.15 eV. All set of calculations were performed at the Γ point within the CP2K package.^[^ ^37^ ^]^

The computed radiative lifetimes (τ_r_) were estimated as the inverse of sum of the Einstein's coefficients (A_i_), which represent the spontaneous transition probabilities from the ground (S_0_) to the i‐excited states (S_i_), by following the procedure previously reported.^[^ ^38^ ^]^ These coefficients can be estimated as:

(1) $$A_{i}=\frac{4\omega_{i}^{3}}{3\hbar^{4}C^{3}}\;\;{\left|\mu_{i}\right|}^{2}$$

where ω_i_ and µ_i_ stand for the exciton energy and the transition dipole moment of the i excited state; whereas *ħ* and *c* represents the reduced Plank and speed of light constants, respectively.

The potential energy surfaces (PES) curves were computed by fixing the Ti(cluster)‐O(ligand) bond distances and relaxing the rest of the atomic positions by adopting the same computational parameters as the ones employed in the previous work.^[^ ^6^ ^]^ These constrained relaxations were conducted within the Broyden–Fletcher–Goldfarb–Shanno (BFGS) algorithm as implemented in CP2K package, till the computed forces reach a maximum value equal to 10^−3^ Hartree per Bohr.^[^ ^39^ ^]^ The energy of the excited states was then estimated by adding the energy gap computed with hybrid functional (PBE0) to the corresponding ground state energy, assuming that the exciton binding energies (i.e., energy difference between optical and electronic gap) remain constant for all explored geometries. Notably this assumption was found valid when computing the PES curves associated to the main vibrational modes of layered lead‐free double perovskites, as estimated by means of Bethe‐Salpeter Equation (BSE) excited state calculations, which were performed to compute the exciton binding energies.^[^ ^40^ ^]^

### Activity Measurements

MIP‐177(Ti)‐LT and MIP‐177(Ti)‐HT were evaluated as heterogeneous photocatalysts for the hydrogen evolution reaction (HER). Briefly, 5 mg of the MOF were dispersed in a mixture of H_2_O (8 mL) and MeOH (2 mL) in a quartz reactor (51 mL). The system was sonicated for 10 min (450 W) to improve homogeneity. Afterwards, the quartz reactor was purged with an argon flow (20 min) to remove oxygen and finally filled with argon to a pressure of 2 bars. The photocatalytic reaction was carried out by irradiating the suspension with simulated sunlight (LC‐8 light guide lamp; 1.5 AM filter, 300 mW cm^−2^) from the top of the reactor. Gaseous samples were taken at 2, 4, 6, and 21 h and analyzed via gas chromatography. Before each sampling, the closed reactor was treated with sonication to potentially release trapped H_2_ from within the MOF pores.

## Conflict of Interest

The authors declare no conflict of interest.

## Supporting information



Supporting Information

## Data Availability

The data that support the findings of this study are available from the corresponding author upon reasonable request.

## References

[1] S. Navalón , A. Dhakshinamoorthy , M. Álvaro , B. Ferrer , H. García , Chem. Rev. 2023, 123, 445. 36503233 10.1021/acs.chemrev.2c00460 PMC9837824

[2] Y. Wang , H. Lv , E. S. Grape , C. A. Gaggioli , A. Tayal , A. Dharanipragada , T. Willhammar , A. K. Inge , X. Zou , B. Liu , Z. Huang , J. Am. Chem. Soc. 2021, 143, 6333. 33900747 10.1021/jacs.1c01764 PMC8297731

[3] A. B. Andreeva , K. N. Le , L. Chen , M. E. Kellman , C. H. Hendon , C. K. Brozek , J. Am. Chem. Soc. 2020, 142, 19291. 33119281 10.1021/jacs.0c09499

[4] Y. Paz , J. Phys.: Condens. Matter 2019, 31, 503004. 31469092 10.1088/1361-648X/ab3eda

[5] E. S. Grape , A. M. Davenport , C. K. Brozek , Dalton Trans. 2024, 53, 1935. 38226850 10.1039/d3dt04164f

[6] S. Wang , T. Kitao , N. Guillou , M. Wahiduzzaman , C. Martineau‐Corcos , F. Nouar , A. Tissot , L. Binet , N. Ramsahye , S. Devautour‐Vinot , S. Kitagawa , S. Seki , Y. Tsutsui , V. Briois , N. Steunou , G. Maurin , T. Uemura , C. Serre , Nat. Commun. 2018, 9, 1660. 29695794 10.1038/s41467-018-04034-w PMC5916937

[7] H. Assi , G. Mouchaham , N. Steunou , T. Devic , C. Serre , Chem. Soc. Rev. 2017, 46, 3431. 28537319 10.1039/c7cs00001d

[8] N. Sinha , O. S. Wenger , J. Am. Chem. Soc. 2023, 145, 4903. 36808978 10.1021/jacs.2c13432 PMC9999427

[9] S. Rojas , J. García‐González , P. Salcedo‐Abraira , I. Rincón , J. Castells‐Gil , N. M. Padial , C. Marti‐Gastaldo , P. Horcajada , Sci. Rep. 2022, 12, 14513. 36008470 10.1038/s41598-022-18590-1 PMC9411604

[10] A. G. Baldoví , R. Del Angel , G. Mouchaham , S. Liu , D. Fan , G. Maurin , S. Navalón , C. Serre , H. Garcia , Energy Environ. Sci. 2023, 16, 167.

[11] S. Nandi , S. Wang , M. Wahiduzzaman , V. Yadav , K. Taksande , G. Maurin , C. Serre , S. Devautour‐Vinot , ACS Appl. Mater. Interfaces 2021, 13, 20194. 33885276 10.1021/acsami.1c03644

[12] R. V. Pinto , S. Wang , S. R. Tavares , J. Pires , F. Antunes , A. Vimont , G. Clet , M. Daturi , G. Maurin , C. Serre , Angew. Chem., Int. Ed. 2020, 59, 5135. 10.1002/anie.201913135 31951064

[13] M. Wahiduzzaman , S. Wang , J. Schnee , A. Vimont , V. Ortiz , P. Georges Yot , R. Retoux , M. Daturi , J. S. Lee , J.‐S. Chang , C. Serre , G. Maurin , S. Devautour‐Vinot , ACS Sustainable Chem. Eng. 2019, 7, 5776.

[14] D. C. L. Vasconcelos , V. C. Costa , E. H. M. Nunes , A. C. S. Sabioni , M. Gasparon , W. L. Vasconcelos , Sci. Appl. 2011, 2, 1375.

[15] C. C. Sutton , G. da Silva , G. V. Franks , Chem. ‐ Eur. J. 2015, 21, 6801. 25753376 10.1002/chem.201406516

[16] V. Zeleňák , Z. Vargová , K. Györyová , Spectrochim. Acta, Part A 2007, 66, 262. 10.1016/j.saa.2006.02.050 16829167

[17] O. Ramon , E. Kesselman , R. Berkovici , Y. Cohen , Y. Paz , Polym. Sci., Part B: Polym. Phys. 2001, 39, 1665.

[18] J. Tang , J. R. Durrant , D. R. Klug , J. Am. Chem. Soc. 2008, 130, 13885. 18817387 10.1021/ja8034637

[19] L. M. Peter , A. B. Walker , T. Bein , A. G. Hufnagel , I. Kondofersky , J. Electroanal. Chem. 2020, 872, 114234.

[20] A. Yamakata , T. A. Ishibashi , H. Onishi , Chem. Phys. Lett. 2001, 333, 271.

[21] A. Yamakata , T. A. Ishibashi , H. Onishi , J. Mol. Catal. A 2003, 199, 85.

[22] A. Ben‐Refael , I. Benisti , Y. Paz , Catal. Today 2020, 340, 97.

[23] J. L. Boonem , G. Cantwell , M. D. Shaw , J. Appl. Phys. 1985, 58, 2296.

[24] J. Almond , P. Sugumaar , M. N. Wenzel , G. Hill , C. Wallis , e‐Polym. 2020, 20, 369.

[25] Q. Ye , D. R. Cairnie , D. Troya , N. Kumar , X. Yang , A. J. Morris , J. Am. Chem. Soc. 2023, 146, 101. 38150536 10.1021/jacs.3c12217 PMC10785796

[26] C. Katan , N. Mercier , J. Even , Chem. Rev. 2019, 119, 3140. 30638375 10.1021/acs.chemrev.8b00417

[27] M. Kasha , Discuss. Faraday Soc. 1950, 9, 14.

[28] M. A. Syzgantseva , N. F. Stepanov , O. A. Syzgantseva , J. Phys. Chem. C 2020, 124, 24372.

[29] I. Benisti , Y. Paz , J. Electrochem. Soc. 2019, 166, H3257.

[30] A. Šuligoj , D. Grinberg , Y. Paz , J. Phys. Chem. C 2021, 125, 51.

[31] J. VandeVondele , J. Hutter , J. Chem. Phys. 2007, 127. 10.1063/1.2770708 17887826

[32] S. Goedecker , M. Teter , J. Hutter , Phys. Rev. B: Condens Matter Mater. Phys. 1996, 54, 1703. 10.1103/physrevb.54.1703 9986014

[33] G. Stefan , J. Comput. Chem. 2006, 27, 1787. 16955487 10.1002/jcc.20495

[34] M. Iannuzzi , T. Chassaing , T. Wallman , J. Hutter , Chimia 2005, 59, 499.

[35] M. A. Syzgantseva , N. F. Stepanov , O. A. Syzgantseva , J. Phys. Chem. Lett. 2019, 10, 5041. 31411032 10.1021/acs.jpclett.9b02051

[36] M. A. Syzgantseva , N. F. Stepanov , O. A. Syzgantseva , J. Phys. Chem. C 2020, 124, 24372.

[37] J. Hutter , M. Iannuzzi , F. Schiffmann , J. Vandevondele , W. Interdiscip , Rev. Comput. Mol. Sci. 2014, 4, 15.

[38] M. A. Syzgantseva , N. F. Stepanov , O. A. Syzgantseva , J. Phys. Chem. C 2020, 124, 24372.

[39] R. H. Byrd , P. Lu , J. Nocedal , C. Zhu , J. Sci. Comput. 1995, 16, 1190.

[40] M. Pantaler , V. Diez‐Cabanes , V. I. E. Queloz , A. Sutanto , P. A. Schouwink , M. Pastore , I. García‐Benito , M. K. Nazeeruddin , D. Beljonne , D. C. Lupascu , C. Quarti , G. Grancini , J. Am. Chem. Soc. Au 2022, 2, 136. 10.1021/jacsau.1c00429 PMC8791057 35098230

