# Supplementary material for: Excited State Transient Phenomena in Two Different Phases of the Photoactive MOF MIP‐177(Ti)
Source: Small. 2025 Feb 19;21(12):2407273. doi: 10.1002/smll.202407273 (PMC11947516; doi:10.1002/smll.202407273)
Supplement: Supplementary file 1 — Supporting Information [file SMLL-21-2407273-s001.docx]

**Supplementary material**

**Excited state transient phenomena in two different phases of the photoactive MOF MIP-177(Ti)**

Aneek Kuila^1^, Valentin Diez-Cabanes^2^, Arianna Melillo^3^, Jonas Gosch^4^, Amarajothi Dhakshinamoorthy^5^, Shilin Yao^6^, Georges Mouchaham^3^, Christian Serre^3^, Hermenegildo García^4^, Sergio Navalon^5^, James R. Durrant^6^, Guillaume Maurin^2^, Yaron Paz^*1^

**Figure S1**: SEM images of the MOFs under studies. A) MIP-177(Ti)-LT B) MIP-177(Ti)-HT. The morphology and the average dimension distribution are in agreement with previously reported data.

**
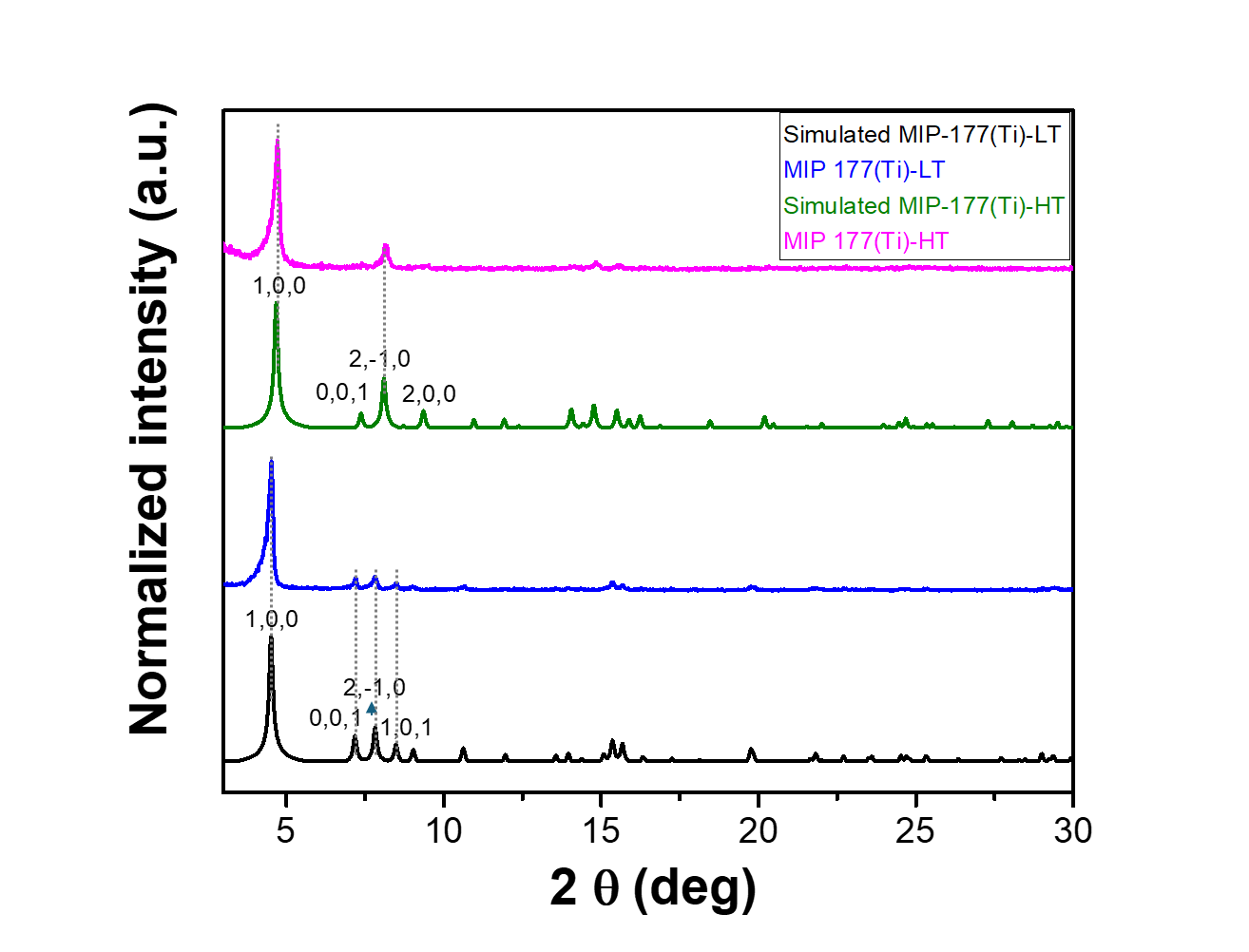
**

**Figure S2**: PXRD patterns (CuKα radiation (λ= 1.5418 Å)) of MIP-177(Ti)-LT (blue) and MIP-177(Ti)-HT (magenta) in comparison with the simulated patterns. The numbers indicated in the figure represent the Miller indices (h, k, l) of the main reflections.


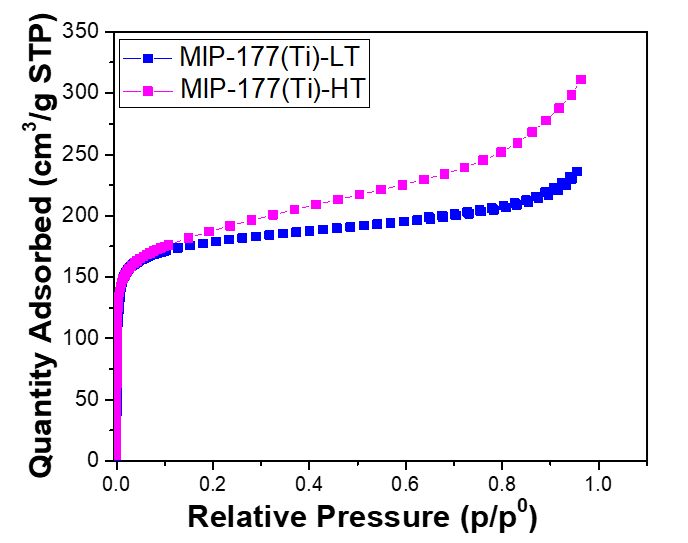


**Figure S3**: N_2_ sorption isotherms at 77K. Calculated SBET are 698 and 691 m^2^/g for MIP-177(Ti)-HT and MIP-177(Ti)-LT, respectively.

**





**

**Figure S4:** XPS spectra for C 1s (A), O 1s (B) and Ti 2p (C) of MIP-177(Ti)-LT.

**





**

**Figure S5:** XPS spectra for C 1s (A), O 1s (B) and Ti 2p (C) of MIP-177(Ti)-HT.







**Figure S6**: XPS HOCO regions for MIP-177(Ti)-LT (A) and MIP-177(Ti)-HT (B).

**



**

**Figure S7:** Diffuse reflectance UV-vis spectra (A) and Tauc plot graph (B) of MIP-177(Ti)-LT (black) and MIP-177(Ti)-HT (Red), as indicated.

**Figure S8:** It should be noted that transient measurements performed with KBr pellets exposed to methanol, in the absence of any MOF, did not yield any transient signal in the region of 600-4000 cm^-1^, let alone in the region of 1900-3200 cm^-1^

**
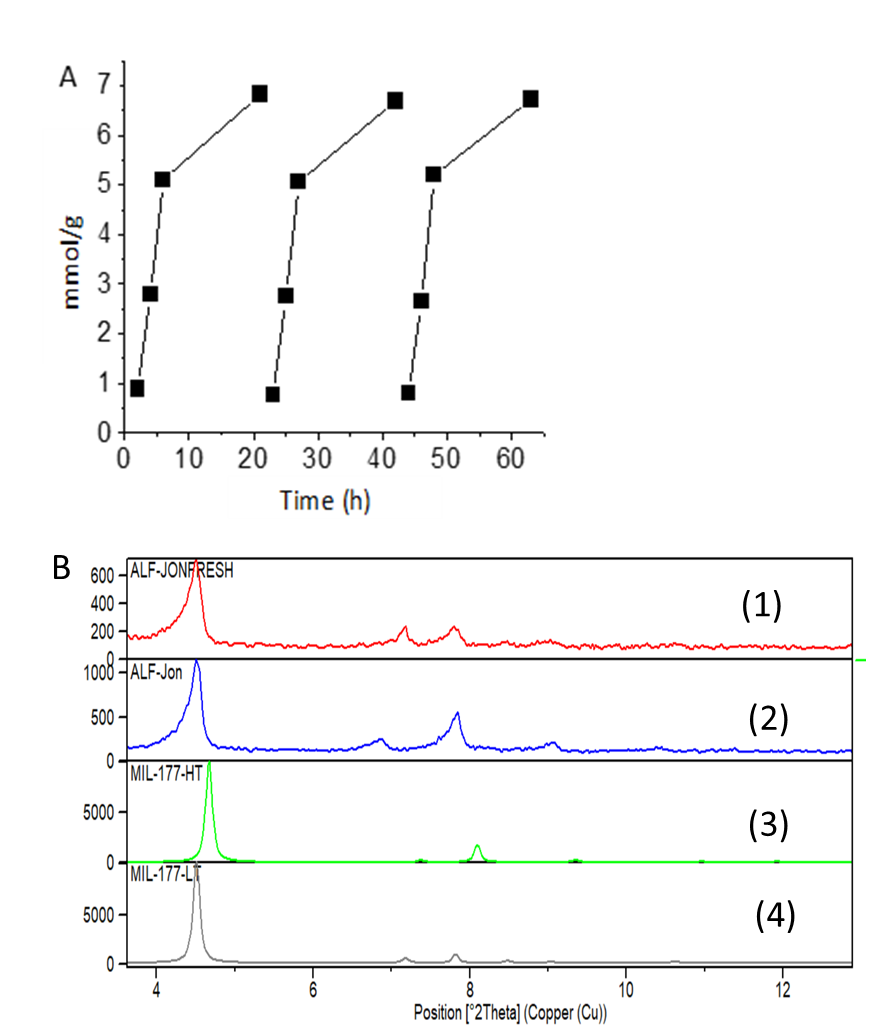
**

**Figure S9**. A. reusability study of the MIP-177(Ti)-LT under simulated solar irradiation, showing same hydrogen evolution kinetics. B. PXRD analysis of the used MIP-177(Ti)-LT, showing that the LT structure was not altered during reaction.: (1) Fresh sample (2) used MIP-177(Ti)-LT, (3) Simulated PXRD pattern of MIP-177(Ti)-HT (4) Simulated PXRD pattern of MIP-177(Ti)-LT. The small difference between (1) and (2) at 2θ of 7° is ascribed to the release of some formate during the photocatalytic studies.


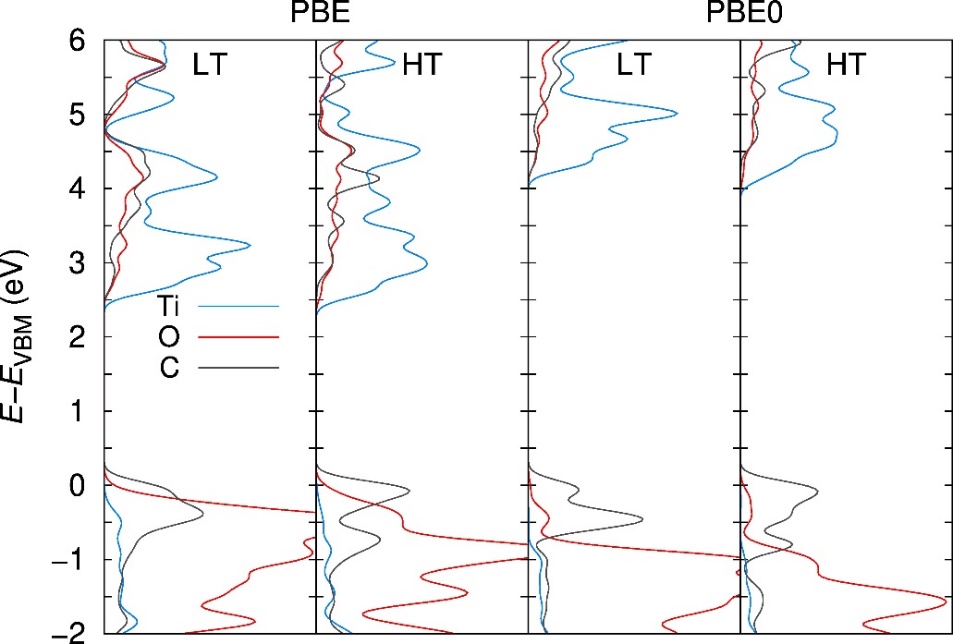


**Figure S10:** Projected density of states (PDOS) of the atoms conforming the MIP-177(Ti)-LT (left) and MIP-177(Ti)-HT structures, as calculated by employing pure (PBE, left) and hybrid (PBE0, right panels) functionals. The valence band maximum (VBM) energy was set as reference.


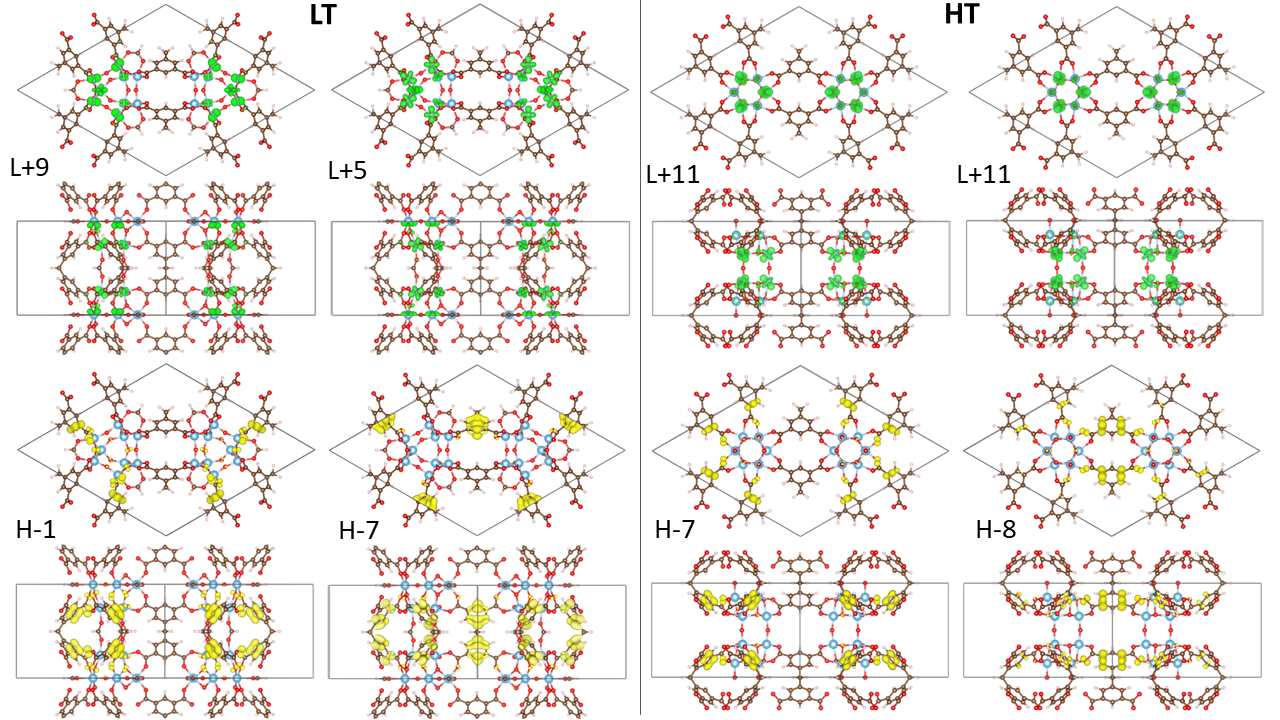


**Figure S11:** Top and lateral views of the shapes for the occupied (yellow) and virtual (green) crystalline orbitals involved in the main transitions of the excited states collected in Table S1 for MIP-177(Ti)-LT (left) and MIP-177(Ti)-HT (right part) compounds. The iso-value used to plot the iso-surfaces was set to 0.03 a.u.


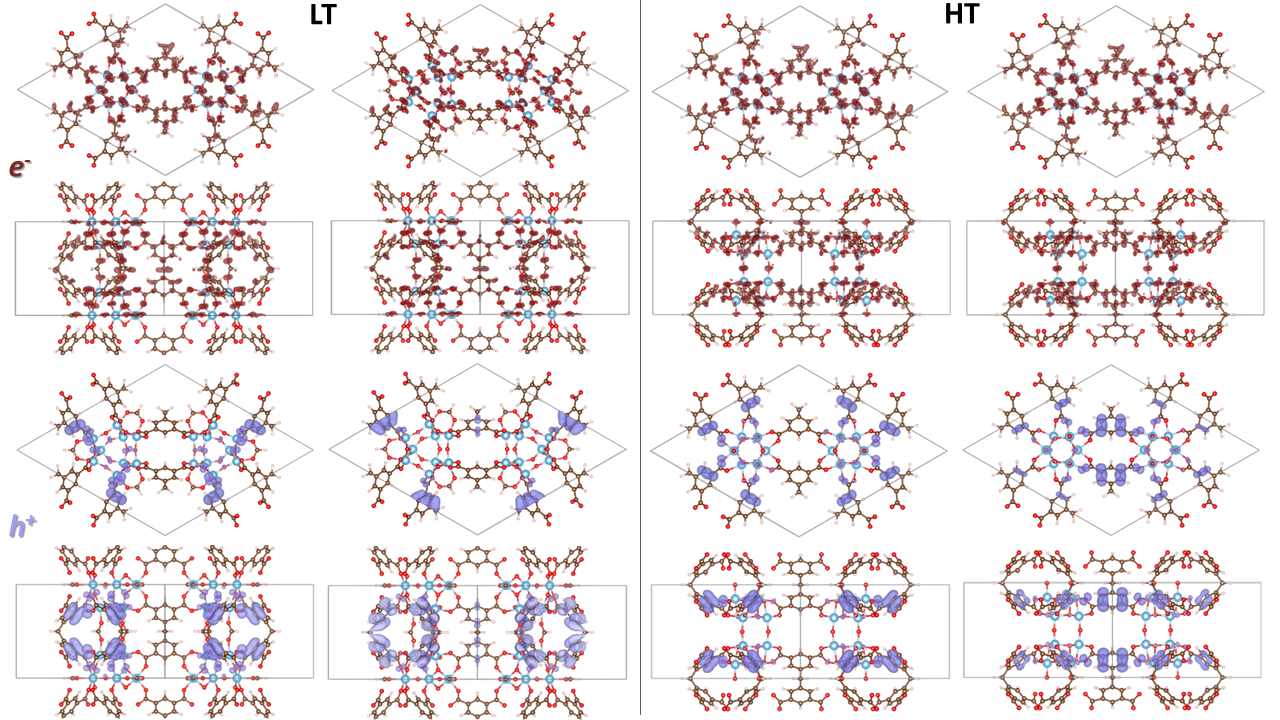


**Figure S12:** Top and lateral views of the shapes for the occupied (purple) and virtual (red) NTOs corresponding to the excited states collected in Table S1 for MIP-177(Ti)-LT (left) and MIP-177(Ti)-HT (right part) compounds. The isovalue used to plot the isosurfaces was set to 0.02 a.u.

**Table S1:** Excited state properties: state number (n), exciton energy (Ex), oscillator strength (f) and main occupied→ virtual crystalline orbital transitions with their corresponding weight (Ci); for the main states employed to build the spectra represented in Figure 15B of the main text.

|  | *n* | *E_x_* (eV) | *f* (10^-2^ a.u.) | *occ*→ *virt* | *C_i_* |
| --- | --- | --- | --- | --- | --- |
| LT | 79 | 2.90 | 5.51 | H-1→L+9 | 0.67 |
|  | 83 | 2.91 | 7.73 | H-7→L+5 | 0.74 |
| HT | 180 | 2.99 | 12.63 | H-7→L+11 | 1.00 |
|  | 181 | 2.99 | 12.42 | H-8→L+11 | 1.00 |

**Table S2:** Vertical transition probability characteristics for MIP-177(Ti)-LT (black) and MIP-177(Ti)-HT (red) phases: state number (n), exciton energy (E_x_), transition dipole moment magnitude (μ_i_), Einstein’s coefficients (A_i_), and radiative recombination lifetimes (τ_r_).

|  | MIP-177(Ti)-LT | | | | MIP-177(Ti)-HT | | | |
| --- | --- | --- | --- | --- | --- | --- | --- | --- |
| *n* | *E_x_* (eV) | *μ*_i_ (D) | A_i_ (s^-1^) | *τ_r_* (s) | *E_x_* (eV) | *μ*_i_ (D) | A_i_ (s^-1^) | *τ_r_* (s) |
| 1 | 2.638 | 9.96E-05 | 0.03 | 33.30 | 2.562 | 1.96E-03 | 10.60 | 0.09 |
| 2 | 2.639 | 4.68E-06 | 6.64E-05 | 1.51E+04 | 2.568 | 1.49E-03 | 6.21 | 0.16 |
| 3 | 2.643 | 8.53E-07 | 2.21E-06 | 4.53E+05 | 2.569 | 1.20E-03 | 3.99 | 0.25 |
| 4 | 2.655 | 1.37 | 5.81E+06 | 1.72E-07 | 2.572 | 0.07 | 1.22E+04 | 8.17E-05 |
| 5 | 2.656 | 1.34 | 5.52E+06 | 1.81E-07 | 2.577 | 0.50 | 6.91E+05 | 1.45E-06 |
| 6 | 2.661 | 0.36 | 3.98E+05 | 2.51E-06 | 2.578 | 0.48 | 6.62E+05 | 1.51E-06 |
| 7 | 2.666 | 1.18E-05 | 4.37E-04 | 2.29E+03 | 2.597 | 0.03 | 2.84E+03 | 3.53E-04 |
| 8 | 2.667 | 0.18 | 1.05E+05 | 9.50E-06 | 2.598 | 0.03 | 3.13E+03 | 3.19E-04 |
| 9 | 2.671 | 0.42 | 5.65E+05 | 1.77E-06 | 2.606 | 1.94E-03 | 11.00 | 0.09 |
| 10 | 2.685 | 0.30 | 2.78E+05 | 3.60E-06 | 2.607 | 1.48E-03 | 6.38 | 0.16 |

**
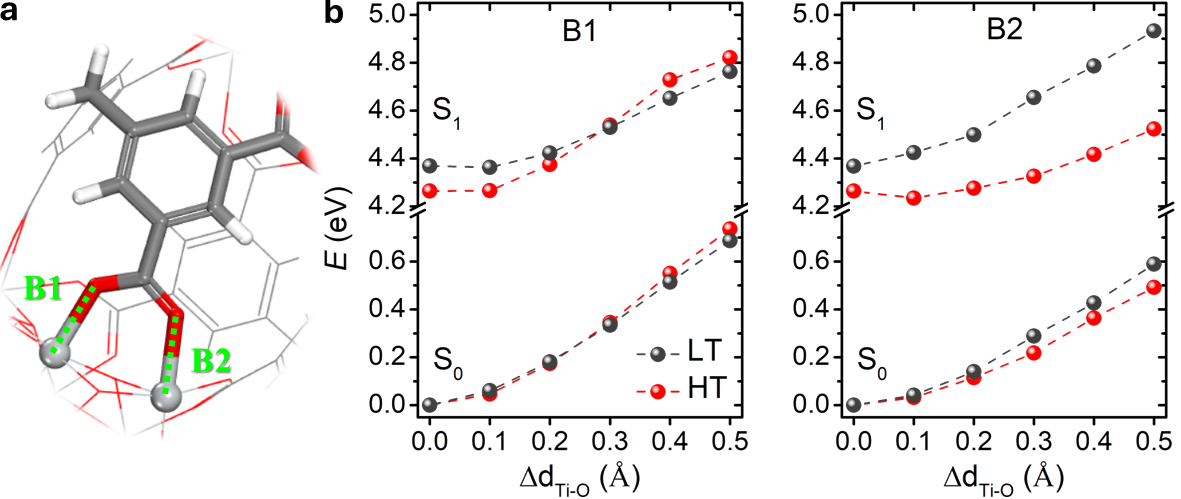
**

**Figure S13:** a) Perspective view of one mdpi ligand grafted to the Ti-cluster highlighting the two types of Ti(cluster)-O(ligand) bonds considered in the PES curves (B1 and B2) by means of dashed green lines; and b) PES curves for the ground (S_0_) and first exited state (S_1_) as a function of the stretching of B1 (left) and B2(right) bonds from their respective equilibrium distance (Δd_Ti-O_).

**Figure S14:** Time-dependent intensity variation of specific peaks in MIP-177(Ti)-LT following 355 nm excitation, A. 509 cm^-1^, B. 656 cm^-1^, C. 1580 cm^-1^, B. 1780 cm^-1^.
